# Methodologies and clinical applications of lower limb muscle ultrasound in critically ill patients: a systematic review and meta-analysis

**DOI:** 10.1186/s13613-024-01395-y

**Published:** 2024-10-24

**Authors:** Roberto Venco, Alessandro Artale, Paolo Formenti, Cristian Deana, Giovanni Mistraletti, Michele Umbrello

**Affiliations:** 1 Dipartimento di fisiopatologia medico-chirurgica e dei Trapianti, Università degli Studi di Milano, Milano, Italy; 2 SC Anestesia, Rianimazione e Terapia Intensiva, Ospedale E. Bassini, ASST Nord Milano, Cinisello Balsamo, Italy; 3 Department of Anesthesia and Intensive Care, Health Integrated Agency of Friuli Centrale, Udine, Italy; 4 SC Rianimazione e Anestesia, Ospedale Civile di Legnano, ASST Ovest Milanese, Via Giovanni Paolo II, 20025 Legnano, MI Italy

**Keywords:** Muscle ultrasound, Critically ill, Lean body mass, Meta-analysis, Muscle wasting, Ultrasound, Systematic review

## Abstract

**Background:**

Reduced muscle mass upon admission and development of muscle wasting are frequent in critically ill patients, and linked to unfavorable outcomes. Muscle ultrasound is a promising instrument for evaluating muscle mass. We summarized the findings of lower limb muscle ultrasound values and investigated how the muscle ultrasound parameters of the examination or the patient characteristics influence the results.

**Methods:**

Systematic review and meta-analysis of studies of lower limb ultrasound critically ill adults. PubMed, CINAHL, Embase, PEDro and Web of Science were searched. PRISMA guidelines were followed, and studies evaluated with the appropriate NIH quality assessment tool. A meta-analysis was conducted to compare the values at admission, short and long follow-up during ICU stay, and the association between baseline values and patient characteristics or ultrasound parameters was investigated with a meta-regression.

**Results:**

Sixty-six studies (3839 patients) were included. The main muscles investigated were rectus femoris cross-sectional area (RF-CSA, *n* = 33/66), quadriceps muscle layer thickness (*n* = 32/66), and rectus femoris thickness (*n* = 19/66). Significant differences were found in the anatomical landmark and ultrasound settings. At ICU admission, RF-CSA ranged from 1.1 [0.73–1.47] to 6.36 [5.45–7.27] cm^2^ (pooled average 2.83 [2.29–3.37] cm^2^) with high heterogeneity among studies (I^2^ = 98.43%). Higher age, higher BMI, more distal landmark and the use of probe compression were associated with lower baseline muscle mass.

**Conclusions:**

Measurements of muscle mass using ultrasound varied with reference to patient characteristics, patient position, anatomical landmarks used for measurement, and the level of compression applied by the probe; this constrains the external validity of the results and highlights the need for standardization.

**Study registration:**

PROSPERO CRD42023420376.

**Supplementary Information:**

The online version contains supplementary material available at 10.1186/s13613-024-01395-y.

## Introduction

Muscle wasting and atrophy frequently affect critically ill individuals, stemming from a range of contributing factors such as extended periods of bed rest, immobilization, systemic inflammation, and the catabolic stress response [1–3]. Furthermore, several studies consistently indicate that a reduced muscle mass upon admission to the ICU independently predicts adverse outcomes [4, 5].

In the early stage of critical illness, protein derived from skeletal muscle is a major source of energy, as proteolysis provides amino acids that are subsequently used for gluconeogenesis [6]. The resulting imbalance, marked by increased degradation at the expense of protein synthesis, leads to rapid skeletal muscle mass wasting [7], manifesting as muscle loss within the first week of hospitalization, and potentially persisting during recovery [8–10]. The extent of muscle wasting is closely associated with the severity of the disease [11].

In recent years, the importance of body composition is becoming increasingly evident in the intensive care field [12]. Notably, in the assessment of body composition, and specifically in the measurement of muscle mass, muscle ultrasound has emerged as a pivotal methodology [13]. Demonstrating simplicity, reproducibility, reliability, and minimal risk to patients, this method has garnered increasing importance at the bedside. Nevertheless, a key limitation lies in the absence of standardized measurements, rendering the results of various studies challenging to compare [14]. Ultrasonographic assessment of muscle mass combines several parameters to assess muscle size, quality, and function [15].

The most commonly evaluated muscles are those from the lower limb, as they are easily accessible, prone to significant wasting and related to the disability outcome [16]. An increasing amount of scientific work is using this method, and in past years some systematic reviews of the literature have focused on the technical aspects of image acquisition and analysis. Ultrasound was found useful for the assessment of the trajectory of change in peripheral muscle mass and architecture, but at the same time it was pointed out that the main limitation of the studies which used this technique was an insufficient description of important variables that can directly influence the quality and validity of the measurements, such as the lack of detail on image acquisition settings, a significant variability in the position of patients during the examination and the anatomical landmarks used, and the potential necessity for adjusting measurements based on body size [17, 18].

As a result, a universally accepted method for ultrasound assessment of muscle mass in the ICU has not been definitively established. To address this gap, we formulated and executed a systematic literature review and meta-analysis. Our objectives were to summarize the values of lower limb muscle ultrasound values, to describe the most frequently employed image acquisition methodologies and parameters, as well as the primary clinical applications of muscle ultrasound and to investigate the influence of muscle ultrasound parameters of the examination or the patient characteristics on the lower limb muscle ultrasound values.

## Methods

This systematic review was conducted and reported following the Preferred Reporting Items for Systematic Reviews and Meta-Analyses (PRISMA) statement [19]. The study protocol was registered on PROSPERO register for systematic reviews (registration n. CRD42023420376).

### Search strategy and study selection

The following databases were retrieved: Web of Science (via Clarivate Analytics), EMBASE (via Elsevier), Pubmed (via the National Library of Medicine), the Cumulative Index to Nursing and Allied Health Literature (CINAHL) and the Physiotherapy Evidence Database (PEDro). We used the following keywords, combined through the appropriate Boolean operators: (“Critical care” OR “Critical illness” OR “Critically ill” OR “Intensive care” OR “Intensive care unit”) AND (Muscle OR “Muscle mass” OR “Skeletal muscle” OR “Lean tissue” OR “Lean mass” OR “Lean body mass”) AND (“Rectus Femoris” OR “Rectus femoris cross-sectional area” OR “Rectus femoris muscle area” OR “Quadriceps muscle layer thickness” OR Quadriceps OR “quadriceps muscle” OR Thigh) AND (Ultrasonography OR Ultrasound OR Echography).

All the studies published from inception up to April 1st, 2023 were imported into and screened from an Excel spreadsheet expressly designed for the purpose, by two independent authors (AA, RV) in order to assess their adequacy according to the inclusion/exclusion criteria at first by title and abstract and then by reading the full text; authors were blind to each other’s decisions and disagreements were solved by consensus or discussion with a third author (MU). The reference lists of included studies were manually screened in order to detect additional potentially suitable studies, and searching personal libraries of the authors.

## Inclusion and exclusion criteria

We conducted a systematic review of published studies concerning methods and clinical applications of lower limb skeletal muscle ultrasound in critically ill patients.

Studies were eligible if they included: adult critically ill patients admitted to ICU, who underwent muscle ultrasound monitoring of the lower limb during their ICU stay; eligibility was limited to studies that described methods and clinical applications of muscle ultrasound of the lower extremities.

Prospective or retrospective observational cohort studies, non-randomized and randomized controlled trial with full text available in English were considered for inclusion.

Case reports, case series, reviews, meta-analyses, pediatric studies, letters to editor and studies not in English or lacking full text were excluded. Supplementary table s1 displays the inclusion/exclusion criteria.

## Outcomes

The main purpose of the review was to provide a systematic account and description of the principal ultrasound techniques for assessing the muscle mass of the lower limb in critically ill subjects. We summarized the frequency and main characteristics of the ultrasound methods used by the different authors to evaluate muscle mass in critically ill patients, including:1) the muscle selected for assessment, 2) the parameters under investigation (muscle thickness – MT, or cross-sectional area - CSA), 3) the type of probe used, 4) the position of the patient, 5) the anatomical landmark and 6) intra- and inter-operator variability.

We conducted a meta-analysis to compare the mean values of each method at three distinct time points: upon admission, at short-term follow-up (within one week of ICU stay), and at long-term follow-up (after more than one week from admission) during the ICU stay. Eventually, given the significant heterogeneity among the studies, we conducted a meta-regression to explore the relationship of various potential moderators on the ultrasound parameter of interest.

## Data extraction

By using a meticulously structured data extraction spreadsheet, we independently gathered pertinent information from the included studies. The extracted data encompassed details such as authors, year of publication, study setting, design, study objectives, purpose of the muscle ultrasound, inclusion/exclusion criteria, sample size, population baseline demographics, and clinical characteristics (including age, sex, BMI, disease severity scores like APACHE II, SOFA, SAPS II), nutritional risk scores, chosen ultrasound method, and its specifications (muscle selected, parameter investigated – MT or CSA, type of probe, patient position, scan site, degree of compression), as well as values of ultrasound muscle measurements. The data points were extracted and documented separately for all available cohorts.

## Risk of bias assessment

The methodological quality of each included study was independently assessed by two authors using the NIH “Quality Assessment of Controlled Intervention Studies” and the “Quality Assessment Tool for Observational Cohort and Cross-Sectional Studies” [20]. Disagreements were solved by consensus or discussion with a third author. The NIH risk of bias tools consists of 14 criteria for Observational Cohort Studies conceived to aid in attributing an overall quality rating to each study (good, moderate or poor).

### Strategy for data synthesis

We conducted a comprehensive descriptive summary of the results, organizing the main characteristics of the included studies and the ultrasound methods employed by each study into tables. The tables included corresponding values at baseline, short-term follow-up (within the first seven days of ICU stay), and long-term follow-up (after more than one week from admission) during the ICU stay.

Relevant data were summarized using mean/standard deviation for continuous variables and rate/95% confidence interval for binary variables.

Meta-analysis was performed to assess the average value for each ultrasound parameter (MT or CSA) according to the level of measurement, compression (minimum or maximum) and follow up (baseline, short follow-up, long follow-up); for studies in which it was not specified, compression was assumed to be minimal. The values provided by the included studies were used in the meta-analysis regardless of the side.

In a study where measurements from four distinct sonographers were reported individually, we opted to utilize the values obtained from the most experienced sonographer for the purpose of the meta-analysis [21]. Due to the excessive heterogeneity of the measurement level used by the various authors, the meta-analysis was performed by referencing values at two pre-selected levels defined as “mid thigh”, which includes measurements at 1/2 of the distance chosen by the author (1/2 anterior-superior or anterior-inferior iliac spine or superior iliac crest-superior border of the patella distance), and “distal thigh”, which includes those taken at all levels in the lower half of the thigh (2/3 or 3/4 or 3/5 anterior-superior iliac spine-superior border of the patella distance).

Given the high heterogeneity in the values of muscle mass reported among the studies, when a measurement was reported in ten or more studies, a random-effects meta-regression was performed to examine the linear association between baseline values and potential case-mix or technique moderators, i.e. the mean age, percentage of male subjects, BMI of patients included in the studies, the mid-thigh or distal landmark, the extent of probe compression. Moderators with Z statistics with a p-value < 0.05 were considered as their slope was significantly different from zero, then the value of muscle mass might vary according to changes in such moderator variables.

The DerSimonian and Laird random-effects model was used to calculate the overall mean and 95% CI; difference in analysis results were considered statistically significant if p-value < 0.05.

When continuous variables were reported as median and IQR or range, we transformed the data to mean and SD as proposed by Wan et al. to avoid losing data [22]. Heterogeneity was assessed using the Cochran’s Q and the I² statistic. Stata 18.0 (StataCorp, College Station, Texas, USA) was used to perform all the analyses.

## Results

**Fig. 1:**
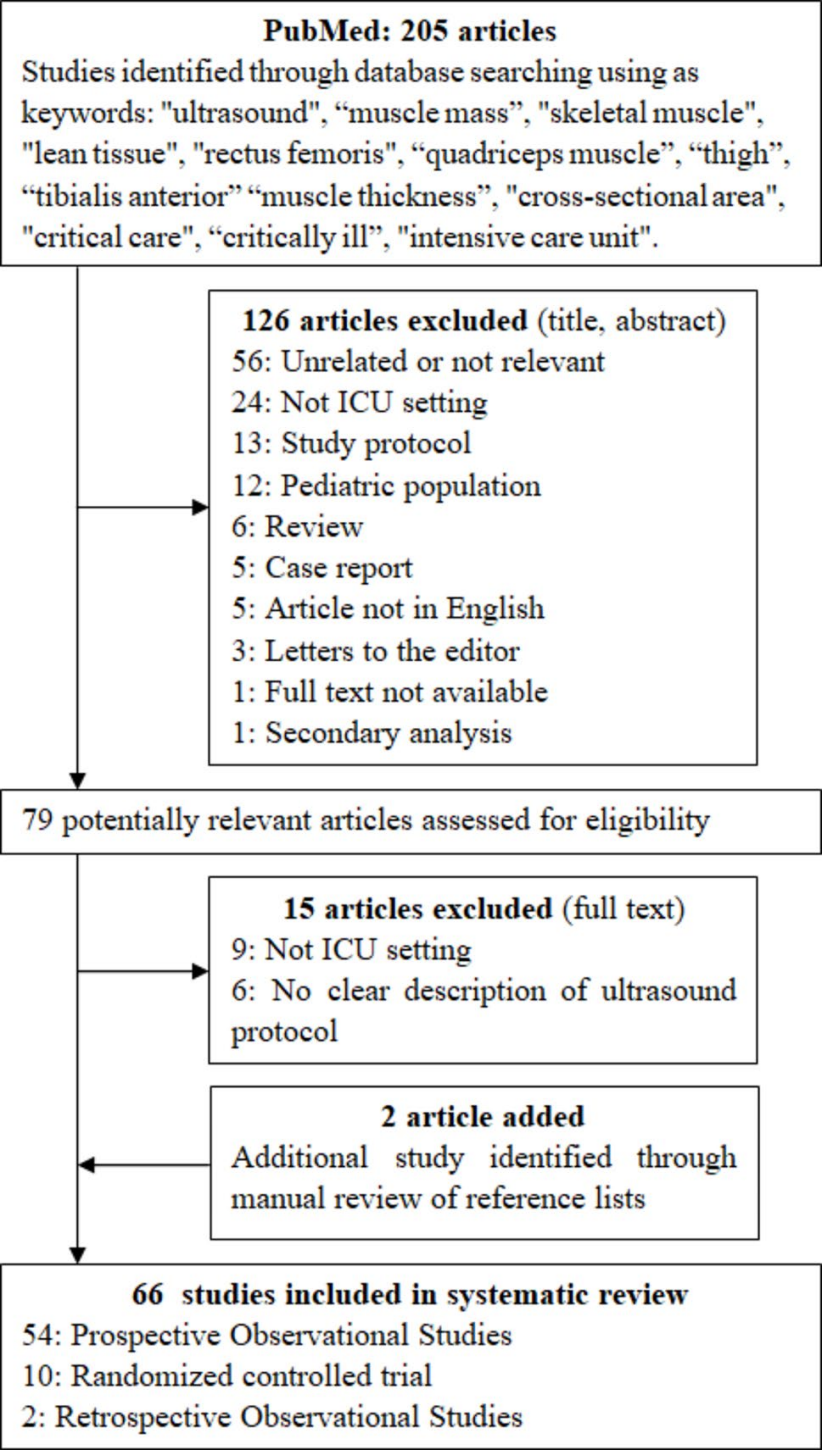
Flow-chart of the study selection process

The literature selection process is reported in Fig. 1. Briefly, the final selection included 66 papers (3839 patients) [9, 16, 21, 23–85], published from 2008 to 2023: 54 prospective observational studies, 10 randomized controlled trials and 2 retrospective observational studies.

Supplementary Tables s2 and s3 summarise the main characteristics of the studies included in the review: the settings and period of enrolment, the inclusion and exclusion criteria, the number of patients enrolled, the aims and main findings of the studies and the ultrasound measurements used. The patients enrolled in the studies ranged from 8 to 284; average age ranged from 30 to 72, the percentage of males varies between 34 and 95%, average SOFA score ranged from 3 to 13 and average APACHE II score from 9 to 67. A progressively growing number of studies over time investigated the use of ultrasound for the assessment and follow-up of muscle mass in critically ill patients; in particular, 52 (78.8%) papers intended to investigate and validate the US method for the assessment of muscle mass and studied the muscle change during ICU stay; the remaining 14 (21.2%) used muscle ultrasound as a method for assessing the outcome of different medical, nutritional or physical interventions.

**Fig. 2:**
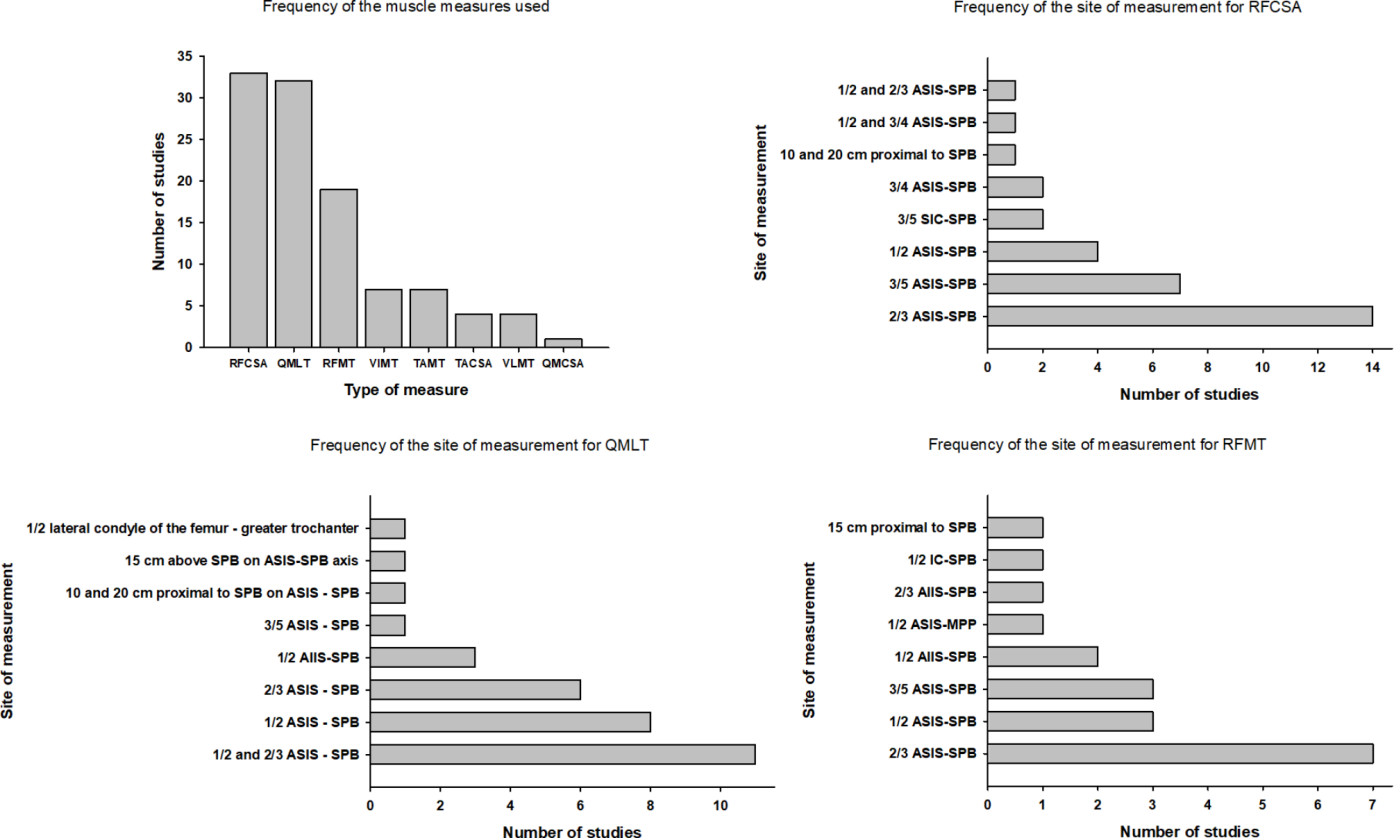
Type and relative frequencies of the ultrasound measurements of muscle mass at the thigh and the landmarks used. The figure shows the different muscle measures used in the studies included in the current systematic review (upper left panel), as well as the anatomical landmarks and sites of measurements selected in the different studies for each of the most frequently used muscle measurements. RFCSA: Rectus Femoris cross sectional area; QMLT: Quadriceps layer muscle thickness; RFMT: Rectus Femoris muscle thickness; VIMT: VastusIntermedius muscle thickness; TAMT: Tibialis Anterior muscle thickness; TACSA: Tibialis Anterior cross sectional area; VLMT: VastusLateralis muscle thickness; QMCSA: Quadriceps muscle cross sectional area; ASIS: anterior superior iliac spine; SIC: superior iliac crest; SPB: superior border patella; SIC: superior iliac crest; MPP: middle point of the patella

As shown in Supplementary Table s2 and in Fig. 2, the muscle mass of the lower limb was evaluated by the different authors using several different measurements at different muscles: Quadriceps muscle layer thickness (QMLT), rectus femoris (RF) thickness and CSA, Vastus intermedius (VI) and Vastus lateralis (VL) thickness, CSA of the entire Quadriceps muscle, Tibialis anterior (TA) thickness and CSA. RF CSA (*n* = 33), QMLT (*n* = 32) and RF thickness (*n* = 19) are the most widely used approaches to assess muscle mass. Twenty-one studies (32%) studies assessed muscle mass at one muscle only, whereas 45 (68%) considered more than one muscle.

Tables 1, 2 and 3, and supplementary tables s4, s5, s6, s7, s8 illustrate the ultrasound protocols (type of transducer, patient position, level and anatomic landmark, compression of the muscle by the probe, side) used by each author for the different measurements and their values at baseline, at a short (within one week of ICU stay) and a longest follow-up during ICU stay (after more than one week from admission). The variability of the muscle measurement used in the different studies, and the specific anatomic landmarks are summarized in Fig. 2.

**Table 1:** Rectus Femoris Cross Sectional Area: summary of ultrasound methods used in each included study for the measurements of RF CSA and its values at baseline, short and long follow-up

Author	Transducer	Patient position	Level	Compression	Side	Baseline (D0-D3) cm^2^	Short FU (≤ D7) cm^2^	Long FU (> D7) cm^2^	Note
Puthucheary et al. 2013 [9]	8 MHz, linear	Supine, rested leg supported in passive extension	3/5 ASIS-SPB	Min	NA	NA	NA	NA	Average of 3 measurements within 10%
Parry et al. [16]	8.5-MHz, linear	Supine, knee in passive extension and neutral rotation	2/3 ASIS-SPB	Min	NA	4.42 ± 0.52	NA	NA	Average of 3 measurements
Mueller et al. [49]	NA	Semisupine (30°-upper body elevation) legs extended and muscles relaxed	3/5 ASIS-SPB	Min	NA	5.90 ± 2.10	NA	NA	Sex-adjusted RFcsa: using published values of healthy adults, RFcsa of females was multiplied by 1.484, whereas males represented the reference group
Annetta et al. [84]	5-7.5 MHz, linear	Supine, both legs in passive extension	3/5 ASIS-SPB	Min	Both	6.20 ± 1.69	5.70 ± 1.16	3.80 ± 1.16	Two measurements in each leg; with lower limb fractures, measurements were taken on the contralateral leg only
Connolly et al. [74]	2–6-MHz, curvilinear	Semisupine, knee supported in passive extension, foot supported in neutral	2/3 ASIS-SPB	Min	Right, unless obstacles	6.36 ± 1.85	5.23 ± 1.88	NA	
Palakshappa et al. [43]	2–5 MHz, curvilinear	NA	2/3 ASIS-SPB	Min	Right unless technical inferiority of the right-sided images	3.98 ± 2.28	NA	NA	Average of 3 measurements within 10%
Hayes et al. [61]	6–15 MHz, linear	Supine with a pillow under their head; hip and knee extended; leg in neutral rotation	2/3 ASIS-SPB	Min	Non-cannulated leg; if both cannulated: that with the venous cannula (vaECMO) or with better thigh access (vvECMO)	4.20 ± 1.30	NA	2.90 ± 1.00	Sagittal plane, average of 3 measurements
Hernández-Socorro et al. [60]	10–12 MHz, linear	Supine with knees extended and relaxed to full extension	2/3 ASIS-SPB	Min	NA	1.10 ± 1.01	NA	NA	
Woo et al. [27]	6–13 MHz, linear	NA	1/2 and 3/4 ASIS-SPB	NA	NA	NA	NA	Right 1/2: 5.28 ± 1.89 Right 3/4: 2.10 ± 0.95 Left 1/2: 5.28 ± 1.89 Left 3/4: 2.19 ± 1.14	
Twose et al. [30]	5 − 2 MHz, curvilinear	Semi-supine with the legs rested in passive extension	2/3 ASIS-SPB	Min	NA	NA	NA	NA	Average of 3 measurements within 10%
Mukhopadhyay et al. [48]	linear	NA	2/3 ASIS-SPB	NA	Both	NA	NA	NA	
Borges et al. [79]	7.5 MHz, linear	Supine, limbs resting on knee extension, after 30’ rest	3/5 SIC-SPB	Min	NA	5.21 ± 1.13	NA	4.37 ± 1.10	Average of 3 measurements within 10%
Borges et al. [80]	7.5 MHz, linear	Supine, limbs resting on knee extension, after 30’ rest	3/5 SIC-SPB	NA	NA	5.11 ± 0.85	4.49 ± 0.84	4.43 ± 0.93	Average of 3 measurements within 10%
Nickels et al. [45]	NA	NA	2/3 ASIS-SPB	NA	Right, unless obstacles	NA	NA	NA	Average of 3 measurements
Pita et al. [39]	Linear	Supine	3/4 ASIS-SPB	NA	Right	1.58 ± 0.67	NA	NA	Average of 3 measurements
Mayer et al. [52]	8.5-MHz, linear	NA	2/3 ASIS-SPB	Min	Right	2.99 ± 0.99	2.47 ± 0.88	NA	Average of 3 measurements
Lee et al. [54]	13 − 6 or 15 − 6 MHz, linear	Supine with leg relaxed, knee in full extension with patella facing the ceiling, in neutral external rotation	2/3 ASIS-SPB	Min	NA	NA	NA	NA	Average of 2 measurements
McNelly et al. [51]	6–15 MHz, linear or 1–5 MHz, curvilinear	Semi-supine with a 30° upward incline at the head	3/5 ASIS-SPB	Min	NA	NA	NA	NA	Average of 3 measurements
Nakanishi et al. [46]	Linear	Supine, knees in passive extension	1/2 ASIS-SPB	Min	NA	NA	NA	NA	
Nakanishi et al. [47]		Supine, passive knee extension	1/2 ASIS-SPB	NA	NA	NA	NA	NA	
Xie et al. [25]	NA	Supine, legs flat, muscles relaxed	3/5 ASIS-SPB	NA	NA	ICU-AW: 2.49 ± 0.78 NON ICU–AW: 2.51 ± 0.66	ICU–AW: 2.04 ± 0.64 NON ICU–AW: 2.34 ± 0.61	NA	
Baston et al. [81]	3–12 MHz, linear	Hip 0–10° flexion, 0–10° abduction, neutral rotation, knee in neutral	10 and 20 cm proximal to SPB along ASIS-SPB	Min	NA	NA	NA	NA	
Umbrello et al. [29]	6–14 MHz, linear	Supine, rested leg supported in passive extension	3/5 ASIS-SPB	Min	Right	Alive: 3.04 ± 1.48 Death: 2.62 ± 1.10	Alive: 2.52 ± 1.14 Death: 1.73 ± 0.58	NA	Average of 3 measurements within 10%
Rodrigues et al. [38]	NA	Supine	1/2 and 2/3 ASIS-SPB	Min	Right	1/2: 5.5 ± 6.0 2/3: 4.1 ± 4.63	1/2: 5.6 ± 4.2 2/3: 3.38 ± 3.9	NA	Average of 2 measurements
Zhang et al. [23]	10–13 MHz, linear	Supine, extended knees and relaxed muscles, toes pointing to the ceiling	2/3 AIIS-SPB	Min	Both	NA	NA	NA	Average of 3 measurements within 10%
Arai et al. [83]	Linear	Supine, knees extended	1/2 ASIS-SPB	Min	Dominant limb or right limb if no information	5.10 ± 2.00	NA	NA	Average of 3 measurements
Hernández-Socorro et al. [60]	10–12 MHz, linear	Supine, knees relaxed and fully extended	2/3 ASIS-SPB	Min	Both	NA	NA	NA	Average of 3 measurements, average between both leg
Passos et al. [73]	1.6–4.6 MHz, convex	NA	1/2 ASIS-SPB	Min	Right	Alive: 7.40 ± 1.80 cm^2^/m^2^ Death: 6.10 ± 1.60 cm^2^/m^2^	NA	NA	Average of 3 measurements. RFcsa corrected by body surface (cm^2^/m^2^)
Formenti et al. [66]	8 MHz, linear	Rested leg supported in passive extension	3/5 ASIS-SPB	NA	NA	1.76 ± 0.83	NA	NA	Average of 3 consecutive measurements within 10%
Formenti et al. [67]	8 MHz, linear	Supine, rested leg supported in passive extension	2/3 ASIS-SPB	Min	Right	2.35 ± 0.90	1.78 ± 0.69	NA	Average of 3 consecutive measurements within 10%
Paolo et al. [42]	8 MHz, linear	Supine, extended knees	2/3 ASIS-SPB	Min	Right	ICU–AW: 2.26 ± 0.85 NON ICU–AW: 2.21 ± 1.15	ICU–AW: 1.60 ± 0.50 NON ICU–AW: 1.70 ± 0.80	NA	Average of 3 consecutive measurements within 10%
Kangalgil et al. [57]	13 − 4 MHz, linear	Supine, knees extended and relaxed	2/3 ASIS-SPB	Min	Right, unless obstacles	4.45 ± 0.99	3.70 ± 0.93	NA	Average of 2 measurements; a third measurement if variation > 10%
Hrdy et al. [58]	3–9 MHz, linear	NA	3/4 ASIS-SPB	Min	At the discretion of the clinician	< 10% decrease RFcsa: 2.33 ± 1.17 ≥ 10% decrease RFcsa: 2.64 ± 1.39	< 10% decrease RFcsa: 1.99 ± 1.19 ≥ 10% decrease RFcsa: 2.36 ± 1.14	NA	Smallest and largest values omitted, average calculated from remaining four values

*Note*: Data reported as mean ± SD, median [IQR]. All measurements are expressed in cm [2]; if studies have expressed data in different units, these are specified in the table

**Table 2:** Quadriceps muscle layer thickness: summary of ultrasound methods employed in each included study for the measurements of QMLT and its values at baseline, short and long follow-up

Author	Transducer	Patient Position	Level	Compression	Side	Baseline (D0-D3) cm	Shortest FU (≤ D7) cm	Longest FU (> D7) cm	Note
Gruther et al. [62]	4–7 MHz, linear	Supine. Legs relaxed lying flat in extension	1/2 and 2/3 ASIS-SPB	NA	Both	NA	NA	NA	average of 2 measurements (measuring points 1/2 and 2/3) on each leg
Baldwin et al. [82]	10 MHz, linear	Supine. Knee extended and leg in neutral abduction and rotation	1/2 ASIS-SPB	NA	Right	NA	NA	1.68 ± 0.61	Average of 3 measurements
Sarwal et al. [21]	8.5-MHz, linear	Supine. Leg supported in passive extension and neutral rotation	2/3 ASIS-SPB	NA	Right	1.82 ± 0.75^A^ 1.80 ± 0.70^B^ 1.80 ± 0.80^C^ 1.71 ± 0.68^D^	NA	NA	average values obtained by four different sonographers (A, B, C, D)
Francis et al. [65]	5–10 MHz, linear	Supine Knees extended, legs in neutral position relaxed	1/2 ASIS-SPB	NA	NA	1.55 ± 0.55	1.32 ± 5.10	NA	
Paris et al. [107]	Linear	Supine. Knees extended and relaxed	1/2 and 2/3 ASIS-SPB	Max	Both	1.30 ± 0.60	NA	NA	Each landmark imaged twice and averaged across each leg and then between legs
Chapple et al. [76]	13 − 6 MHz	Supine. Legs relaxed, lying flat in extension	1/2 and 2/3 ASIS-SPB	Max	Both	1.84 ± 0.71	NA	NA	2 measurements were taken by one researcher; a third measurement was taken if the first two measurements differed by > 10%. The final QMLT was calculated as the means of all measurements
Palakshappa et al. [43]	2–5 MHz, curvilinear	NA	2/3 ASIS-SPB	Min	Right	2.15 ± 1.12	NA	NA	Average of 3 measures within 10%
Pardo et al. [41]	12 MHz, linear	Leg maintained into a lower limb orthosis	1/2 ASIS-SPB	Max	Both	1.72 ± 0.67	1.45 ± 0.56	1.30 ± 0.89	Mean value of the left and right QMLT
Katari et al. [56]	5–10 MHz, Linear	Supine	1/2 AIIS-SPB	Min	Right	2.50 ± 0.79	2.26 ± 0.81	NA	
Hayes et al. [61]	6–15 MHz, linear	Supine. Pillow under their head; hip and knee extended; leg in neutral rotation	2/3 ASIS-SPB	Min	Non-cannulated leg; if both cannulated: that with the venous cannula (vaECMO) or with better thigh access (vvECMO)	2.10 ± 0.60	NA	1.40 ± 0.40	Sagittal plane, average of 3 measures
Martin et al. [7]	13 MHz, linear	Supine. Head of the bed at 30–45°; right leg extended, toes pointing at the ceiling	1/2 AIIS-SPB	Max	Right	1.35 ± NA	NA	NA	Average of 2 measurements; if the difference between the measurements is more than 0.1 cm, a third measurement is taken to calculate the average
Silva et al. [36]	7.5 MHz	NA	1/2 ASIS-SPB	Min	NA	NA	NA	NA	Average of the measurements obtained from the 3 best images
Fetterplace et al. [68]	13 − 6 MHz	Supine. Legs relaxed and extended	1/2 and 2/3 ASIS-SPB	NA	Both	NA	NA	NA	Duplicate measurements were taken at each landmark and the mean of the4 linear thicknesses was calculated for each leg separately
Özdemir et al. [44]	Linear; Convex in obese and edematous patients	Supine. Quadriceps femoris muscle relaxed	1/2 ASIS-SPB	Both	Both	Right MIN 2.10 ± 1.11 Left MIN 2.11 ± 0.96 Right MAX 0.77 ± 0.52 Left MAX 0.78 ± 0.42	NA	NA	Average of 3 measurements
Mayer et al. [52]	8.5 MHz, linear	NA	2/3 ASIS-SPB	Min	Right	2.04 ± 0.71	1.77 ± 0.62	NA	Average of 3 measurements
Fetterplace et al. [69]	13 − 6 MHz	NA	1/2 and 2/3 ASIS-SPB	Both	Both	Max: 1.80 ± 0.60 Min: 3.30 ± 0.80	Max: 1.80 ± 0.60 Min: 3.20 ± 1.00	NA	4 measurements, for minimal and maximal pressures were recorded separately. Where bilateral measurements were available, the average between the two lower limbs was used. If variability > 10%, a third measurement was done
Lee et al. [54]	13 − 6 or 15 − 6 MHz, linear	Supine. Leg relaxed, knee in full extension with patella facing the ceiling, in neutral external rotation	2/3 ASIS-SPB	Min	NA	NA	NA	NA	Average of 2 measurements
Dimopoulos et al. [72]	7.5 MHz, linear	Supine.Right thigh in neutral position. knee extended and muscles relaxed	1/2 AIIS-SPB	Min	NA	2.60 ± 0.72	2.37 ± 0.80	NA	Average of 2 measurements
Tourel et al. [32]	4-12 MHz, linear	Supine. Leg into a foam device maintaining the feet in an upright position	15 cm above SPB on ASIS-SPB axis	Min	Right	NA	NA	NA	
Nakanishi et al. [47]	Linear	Supine. Knees in passive extension	1/2 ASIS-SPB	Min	NA	NA	NA	NA	
Dresen et al. [71]	3–12 MHz, linear	NA	1/2 and 2/3 ASIS-SPB	Max	Both	Intervention: 1,35 ± 0,74 Control: 1,34 ± 0,72	NA	NA	Measurements were conducted twice on both legs; a third measurement was performed if variation > 10%. Average values at each measuring points for each leg; averages of all four QMLT measurement points
Yanagi et al. [24]	3.4–8 MHz	Supine. Knees extended	1/2 ASIS-SPB	Min	NA	NA	NA	2.13 ± 0.84	
Supinski et al. [35]	10 MHz, linear	NA	1/2 lateral condyle of the femur - greater trochanter	NA	Right	Placebo: 2.80 ± 0.96 EPA: 3.3 ± 1.07 HMB: 3.1 ± 1.01 HMB + EPA: 2.6 ± 1.41	NA	NA	Average of the 3 best measurements
Baston et al. [81]	3–12 MHz, linear	Hip in 0–10° of flexion. 0–10° of abduction and neutral rotation. Knee in neutral	10 and 20 cm proximal to SPB along ASIS-SPB	Min	NA	NA	NA	NA	
Bury et al. [78]	4–12 Hz, linear	NA	1/2 and 2/3 ASIS-SPB	Max	Based on accessibility	NA	NA	NA	
Lambell et al. [55]	4–12 MHz, linear	Supine. Head of the bed at 30°; knees extended and relaxed	1/2 and 2/3 ASIS-SPB	Min	Both	One thigh: 155.1 ± 49.2cm^2^; Bilateral thighs 154.8 ± 47.9 cm^2^	NA	NA	The “bilateral thigh thickness” was the average across QMLT of both thighs; for each thigh the value was an average of QMLT at 1/2 and 2/3 site. For each site the value was the average of 3 measurements. The final value was the muscle thickness multiplied by limb length
Rodrigues et al. [38]	NA	Supine	1/2 and 2/3 ASIS-SPB	Min	Right	1/2: 2.96 ± 3.11 2/3: 2.48 ± 2.85	1/2: 2.47 ± 2.28 2/3: 2.33 ± 2.64	NA	Average of 2 measurements
Er et al. [70]	10.7 MHz, linear	Supine. both legs in passive extension	3/5 ASIS-SPB	NA	Right	Weaning: 2.40 ± 0.46 Weaning failure: 1.75 ± 1.14	NA	NA	Average of 3 measurements
Toledo et al. [33]	5-13 MHz, linear	Semi-recumbent position (30–45°). knees extended	2/3 ASIS-SPB	Min	Both	Right 2.00 ± 0.91 Left 1.97 ± 0.83	NA	NA	Each landmark was imaged twice and averaged across each leg
Arai et al. [83]	Linear	Supine. knees extended	1/2 ASIS-SPB	Min	Dominant limb or right limb under no information	2.43 ± 0.98	NA	NA	Average of 3 measurements
Anand et al. [85]	6–12 MHz, linear	Supine. Legs relaxed lying flat in extension	1/2 and 2/3 ASIS-SPB	NA	Both	1.95 ± 0.50	1.80 ± 0.63	NA	measurements at the 4 points were averaged to a single value; 3 such values were averaged for final QMLT
Chapple et al. [75]	5–13 MHz	Supine Legs relaxed lying flat in extension, toes facing the roof	1/2 and 2/3 ASIS-SPB	Min	Right, unless obstacles	Standard EN 2.86 ± 1.30 Energy EN 3.07 ± 1.17	Standard EN 2.38 ± 1.08 Energy EN 2.68 ± 0.72	Standard EN 2.39 ± 1.06 Energy EN 2.90 ± 1.27	Average of the values at the two measuring point for each site, plus a third if there was a variability > 10% between the two

*Note*: Data reported as mean ± SD, median [IQR]. All measurements are expressed in cm; if studies have expressed data in different units, these are specified in the table

Abbreviations: AIIS: anterior inferior iliac spine; ASIS: anterior superior iliac spine; D followed by a number refers to days of follow up; FU: follow-up; ICU: intensive care unit; Max: maximum; Min: minimum; NA: not available; QMLT: quadriceps layer muscle thickness; SPB: superior patella border

**Table 3:** Rectus Femoris thickness: summary of ultrasound methods employed in each included study for the measurements of RF-Thick and its values at baseline, short and long follow-up

Author	Transducer	Patient Position	Level	Compression	Side	Baseline (D0-D3) cm	Short FU (≤ D7) cm	Long FU (> D7) cm	Note
Gerovasili et al. [63]	7.5 MHz, linear	Supine. Legs lying flat in extension	1/2 ASIS-MPP	Min	Both	EMS: Right 1.42 ± 0.48 Left 1.34 ± 0.39 Control: Right 1.59 ± 0.53 Left 1.62 ± 0.55	EMS: Right 1.31 ± 0.45 Left 1.2 ± 0.41 Control: Right 1.37 ± 0.5 Left 1.43 ± 0.6	NA	
Cartwright et al. [77]	18 MHz, linear	NA	15 cm proximal to SPB	Min	NA	2.64 ± NA	3.10 ± NA	2.59 ± NA	
Parry et al. [16]	8.5-MHz linear	Supine. Knee in passive extension and neutral rotation	2/3 ASIS-SPB	Min	NA	2.44 ± 0.76	NA	NA	Average of 3 measurements
Annetta et al. [84]	5-7.5 MHz, linear	Supine. Both legs in passive extension	3/5 ASIS-SPB	Min	Both	1.73 ± 0.39	1.73 ± 0.39	1.23 ± 0.46	Two measurements in each leg; with lower limb fractures, measurements on the contralateral leg only
Katari et al. [56]	5–10 MHz, Linear	Supine	1/2 AIIS-SPB	Min	Right	1.37 ± 0.41	1.22 ± 0.47	NA	
Hayes et al. [61]	6–15 MHz, linear	Supine. Pillow under the head; hip and knee extended; leg in neutral rotation	2/3 ASIS-SPB	Min	Non-cannulated leg; if both cannulated: that with the venous cannula (vaECMO) or with better thigh access (vvECMO)	1.1 ± 0.3	NA	0.6 ± 0.2	Sagittal plane, average of 3 measures
Hernández-Socorro et al. [59]	10–12 MHz, linear	Supine. Knees extended and relaxed to full extension	2/3 ASIS-SPB	Min	NA	0.59 ± 0.39	NA	NA	Averaged measurements were estimated
Silva et al. [36]	7.5 MHz, linear	NA	1/2 ASIS-SPB	Min	NA	NA	NA	NA	Average value of 3 measurements
Nickels et al. [45]	NA	NA	2/3 ASIS-SPB	NA	Right, unless inaccessible	NA	NA	NA	Average value of 3 measurements
Mayer et al. [72]	8.5 MHz, linear	NA	2/3 ASIS-SPB	Min	Right	0.98 ± 0.3	0.81 ± 0.27	NA	Average of 3 measurements
Dimopoulos et al. [72]	7.5-MHz, linear	Supine. Right thigh in neutral position, knee extended, and muscles relaxed	1/2 AIIS-SBP	Min	Right	1.38 ± 0.37	1.25 ± 0.52	NA	Average of 2 measurements
Er et al. [70]	10.7 MHz, linear	Supine. Legs in passive extension	3/5 ASIS-SPB	NA	Right	Weaning: 1.36 ± 0.37 Weaning failure: 0.95 ± 0.77	NA	NA	Average of 3 measurements
Tanaka et al. [34]	Linear	Supine. Relaxed	1/2 ASIS-SPB	NA	Both, averaged	1.74 ± 0.52	NA	1.14 0.53	Average of RF thickness from both legs
Zhang et al. [23]	10–13 MHz, linear	Supine. Extended knees and relaxed muscles, toes of patients pointing to the ceiling	2/3 AIIS-SPB	Min	Both	NA	NA	NA	Average of 3 measurements within 10%
Hernández-Socorro et al. [60]	10–12 MHz, linear	Supine. Knees relaxed and fully extended	2/3 ASIS-SPB	Min	Both	0.62 ± 0.22	NA	NA	Average of 3 measurements, average between both leg
Mendes et al. [50]	NA	Supine. Knee fully extended, leg relaxed	1/2 IC-SPB	Min	NA	1.46 ± 0.30	1.29 ± 0.31	NA	Average of 3 measurements
Formenti et al. [66]	8 MHz, linear	Pt at 45° degrees, rested leg supported in passive extension	3/5 ASIS-SPB	NA	NA	0.61 ± 0.1	NA	NA	Average of 3 measurements within 10%
Maskos et al. [53]	20 MHz, linear	NA	2/3 ASIS-SPB	Min	Both	1.01 ± 0.26	NA	0.76 ± 0.14	
Wu et al. [26]	8 MHz, Linear	Supine. Right foot extended	1/2 ASIS-SPB	Min	Right	1.28 ± 0.41	NA	NA	

*Note*: Data reported as mean ± SD, median [IQR]. All measurements are expressed in cm; if studies have expressed data in different units, these are specified in the table

Abbreviations: AIIS: anterior inferior iliac spine; ASIS: anterior superior iliac spine; D followed by a number refers to days of follow up; ECMO: extracorporeal membrane oxygenation; EMS: electrical muscle stimulation; FU: follow-up; IC: iliac crest; Max: maximum; Min: minimum; MPP: midpoint of the patella; NA: not available; Pts: patients; SPB: superior patella border

In particular, almost all the authors used linear probes (*n* = 56–84.8% linear probe; *n* = 4–6.1% curvilinear; *n* = 6–9.1% not specified) and patients were examined with leg in passive extension and neutral rotation. Most authors use the minimum compression (*n* = 39–59.1%), while only a few use maximum compression (*n* = 6–9.1%) or both (*n* = 2–3%); 19 authors (28.8%) do not specify the method used.

More specifically, Table 1 summarizes the methods used for the measurements of RF CSA. All the studies uniformly employed minimal compression, with the majority focusing on muscle measurements at the lower part of the thigh (*n* = 26–78.8%). Only a limited number of authors chose to insonate the muscle at the mid-thigh region (*n* = 4–12.1%) or both site (*n* = 3–9.1%). The methods used to assess QMLT are reported in Table 2.

This particular measurement stands out as the only instance where some authors applied maximum compression (*n* = 6–18.8%) or both (*n* = 2–6.2%). Approximately an equal number of studies took measurements at the mid-thigh (*n* = 12–37.5%) or the distal thigh (*n* = 8–25%), while others opted for an average of measurements at these two levels (*n* = 12–37.5%). Details of the methods used for RF thickness are shown in Table 3. Again, all the studies used the minimum compression, a greater number of authors assessed the measurement at mid-thigh (*n* = 7–36.8%), while fewer at distal thigh (*n* = 12–63.2%).

Supplementary Table s9 shows the inter- and intra-operator reliability coefficients estimated for each ultrasound method by the different authors: most of the studies that investigated the reproducibility of ultrasound measurements showed high values of intra- (range 0.74–0.99) and inter-operator (range 0.76–0.99) reliability coefficient.

**Fig. 3:**
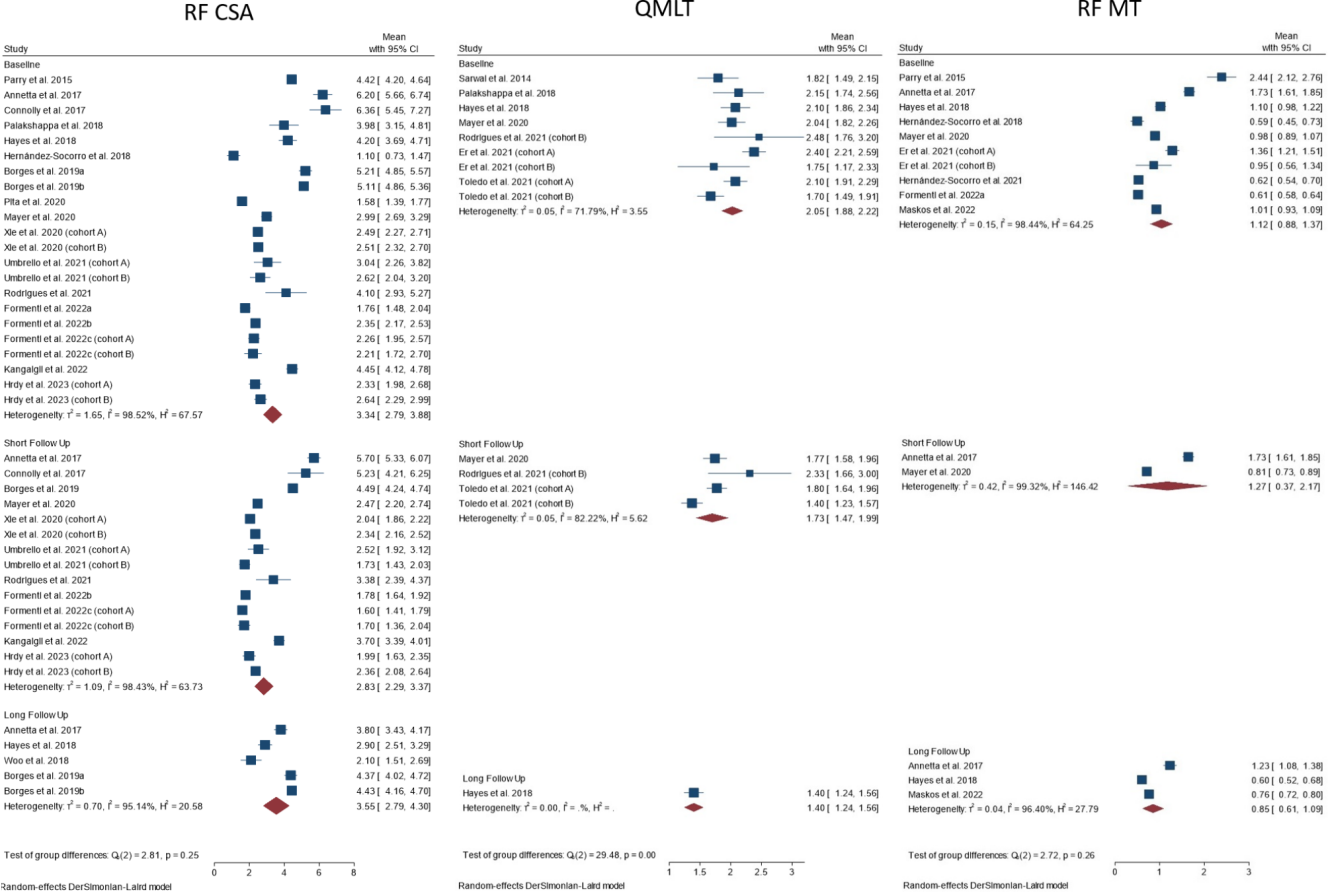
Forest plot of the average value of muscle mass for the three most frequently reported ultrasound methods (Left panel: Rectus femoris CSA, Middle panel: Quadriceps muscle layer thickness, Right panel: Rectus femoris MT) at baseline and at the shortest and longest follow-up. The figure reports the value of muscle mass assessment using no or minimal compression and a distal thigh landmark

A meta-analysis was performed to estimate and compare the values at baseline and at the shortest and longest follow-up for all the ultrasound measurements, and anatomic landmarks. Results for studies using a distal thigh landmark are reported in Fig. 3. In particular, baseline RF CSA values ranged from 1.1 [95%CI 0.73; 1.47] to 6.36 [5.45; 7.27] cm [2], with a pooled estimate of 2.83 [2.29; 3.37] cm [2]and a high heterogeneity among studies (I^2^ = 98.43%). No differences were found among values at baseline, at a short and a long follow-up in the ICU (*p* = 0.25) (Fig. 3, left panel). Similar findings are reported for QMLT (Fig. 3, middle panel) and for RF MT (Fig. 3, right panel). Notably, as far as QMLT is concerned, a significant difference was found between admission and the subsequent follow-up, with pooled estimates of 2.05 [1.88; 2.22] vs. 1.73 [1.47; 1.99] vs. 1.4 [1.24; 1.56] cm, *p* < 0.001. Supplementary figure S1-S3 shows similar results for the analysis of the same muscle groups at a mid-thigh landmark (Supplementary figure S1), or for studies using QMLT and different amount of muscle probe compression (Supplementary figure S2), or for the analysis of other muscles (Supplementary figure S3).

**Fig. 4:**
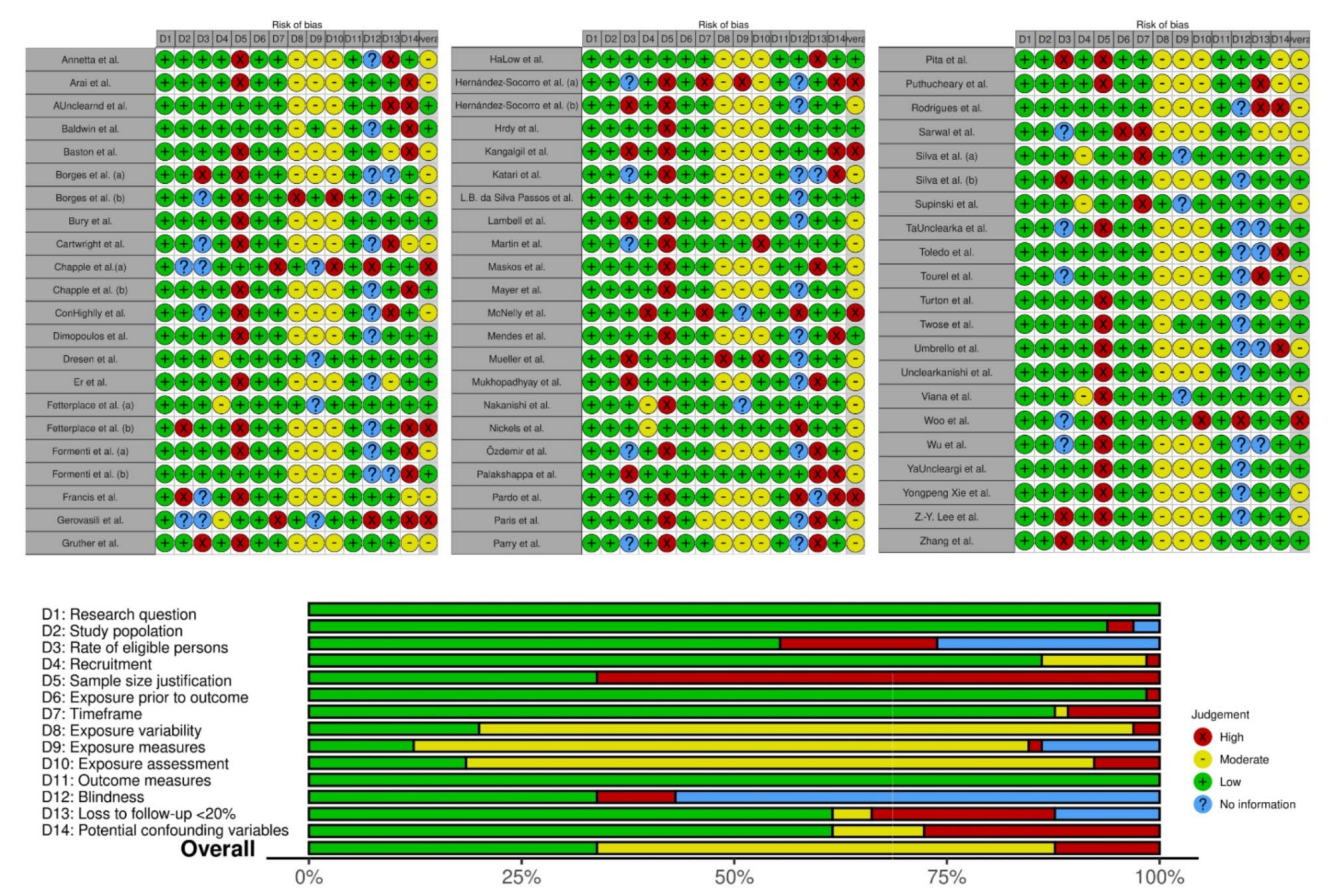
Summary of the risk of bias of the included studies using the appropriate NIH quality assessment tool

Figure 4 reports the summary of the risk of bias of the included studies using the appropriate NIH Quality Assessment tools. Briefly, only 8 (12%) studies were considered at high risk of bias and of low quality, whereas the remaining 58 (88%) had a low or moderate risk of bias and a fair or high quality.

**Fig. 5:**
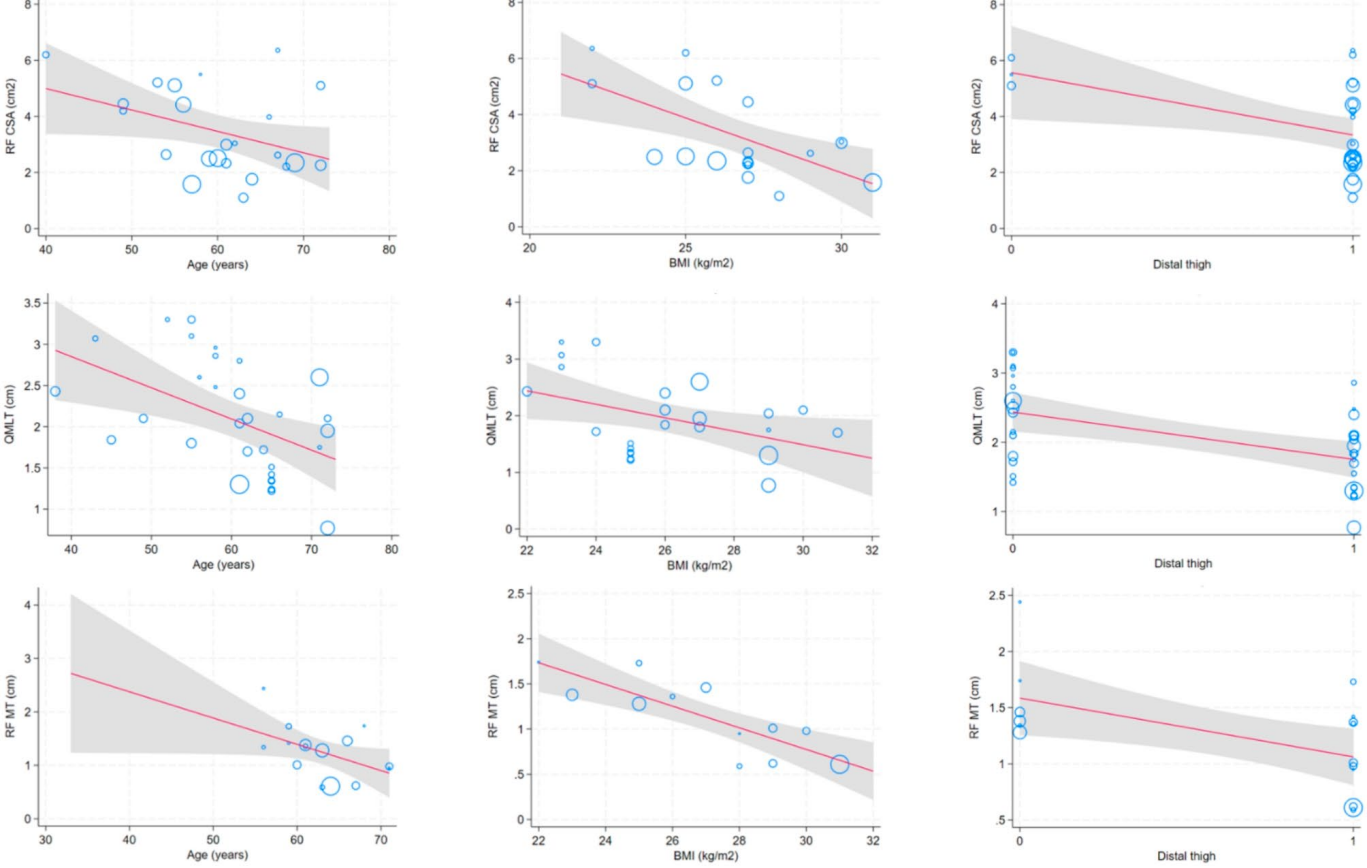
Meta-regression for the crude effects of age (left panel), body-mass index (middle panel) or the use of a distal thigh landmark of subjects included in the studies on baseline RF CSA (upper row), QMLT (middle row) or RF MT (lower row). The point sizes are inversely proportional to the standard error of the mean of the individual studies (i.e., larger/more precise studies are shown as larger circles). Regression line and its prediction intervals (95% CIs) were presented in the figure

In the meta-regression analysis (Fig. 5) a higher age was associated with a reduced baseline RF CSA (coefficient: −0.07, *p* = 0.038); similar results were found for BMI (coefficient: −0.39, *p* = 0.002), and the use of a distal thigh landmark (coefficient: −2.23, *p* = 0.013), whereas the percentage of male subjects included in the studies was not statistically significant (coefficient: 0.01, *p* = 0.574), nor was the severity of the disease, as evaluated by the SAPS II score (coefficient: 0.11, *p* = 0.074). Similar results were found for the effect of the same moderators on QMLT (age: coefficient − 0.37, *p* = 0.003; BMI: coefficient − 0.12, *p* = 0.027; distal landmark: coefficient − 0.68, *p* < 0.001; compression of the probe: coefficient − 0.98, *p* < 0.001) and on RF MT (age: coefficient − 0.05, *p* = 0.025; BMI: coefficient − 0.11, *p* < 0.001; distal landmark: coefficient − 0.52, *p* = 0.013; compression of the probe: coefficient − 0.81, *p* < 0.001).

## Discussion

The main findings of this systematic review and meta-analysis of the literature on the ultrasonographic assessment of lower limb muscles in critically ill subjects can be summarized as follows: (1) the most used methods for the assessment of lower limb muscle mass in critically ill patients are RF CSA, QMLT and RF MT; (2) despite the steadily increasing use, still the measurements performed are widely heterogeneous in terms of patient position, level and anatomic landmark, probe compression; (3) despite the excellent intra- and inter-observer reproducibility, a significant heterogeneity was found in the baseline values reported independently of the measure used, which limited the external validity of the measurements; (4) both factors related to the characteristics of subjects studied (such as age or BMI) and factors related to the technique used (such as the anatomic landmark and probe compression) were significant moderators of the average value of muscle mass found at admission.

The realm of ultrasound as a tool for assessing and monitoring the muscle mass of critically ill patients is rapidly evolving. In recent years, there has been a substantial increase in publications, accompanied by the emergence of several systematic reviews and meta-analyses. However, the majority of these reviews do not specifically concentrate on critically ill subjects [86–89], or they encompass various tools for muscle mass assessment [8] or provide a comprehensive overview of ultrasound use, not limited to the lower limb [17, 90].

A recent systematic review and meta-analysis on adult, community-dwelling or healthy subjects confirmed how ultrasound is a reliable and valid tool for the assessment of muscle size [88]. Similar to the findings presented in the current meta-analysis, the authors found that, despite high methodological quality, the studies were heterogeneous in terms of scanning procedures, which might have influenced the external reliability of the findings. The lack of difference among the pooled mean estimate of muscle mass between baseline measurements and early or late follow-up is apparently counterintuitive, especially in light of the known significant and progressive loss in muscle mass which characterizes critical illness [8, 9]. In general, for a meta-analytic pooled estimate to yield relevant information, two important theoretical assumptions must occur: constituent studies must be characterized by a common mechanism and should aim at the same empirical target [91]. In the present case, however, the large heterogeneity between the findings of the different studies likely depended on the specific measurement protocol (i.e. the lack of a standardization of patient position, level of the scan and probe compression) and differences among the studies (age, body composition, comorbidities and functional status prior to ICU admission of the patients included).

Although a consensus on the optimal method for quantifying muscle mass in critically ill patients is still lacking, it has been demonstrated by other researchers that ultrasound is increasingly emerging as the preferred approach [8]. Despite its acknowledged operator-dependence, studies have affirmed the reliability of ultrasound, particularly in terms of intra- and interobserver correlation [21].We found similar, good values of reliability when summarizing the reproducibility of the data of the studies included in the current review. Moreover, the findings of our systematic review are in line with recent meta-analyses [8], which found how > 80% of studies which investigated muscle mass in critically ill patients used ultrasound, and in particular performed measurements at the rectus femoris and/or quadriceps muscle CSA or MT. The same study highlighted that thickness measurement can significantly underestimate muscle loss compared with CSA, as already shown [92].

The heterogeneity observed in ultrasound measurements of muscle mass values may either signify true variations or be attributed to the specific protocols employed for image acquisition in each study. It is well known that muscle mass decreases with age, approximately 5% per decade after 30 years, up to about 2% annually after the age of 60 [93]. Moreover, independently of age, a variety of comorbid medical conditions was shown to act as independent predictors of lower muscle mass [94]. Taken together, differences in the case-mix among the studies included might imply a different muscle mass among individuals, as shown by the significant, negative, linear relationship found for both age and BMI on RF CSA, QMLT and RF MT. Quite surprisingly, we found no or only a partial change in the absolute values of muscle size across the entire cohort: this may be due to the fact that patients do not exclusively lose muscle fibers, and some degree of ultrasonographic increase in muscle size has been found, presumably as a function of edema formation [95].

On the other hand, independently of true differences in muscle mass, different ultrasound protocols might lead to different results even in the presence of a similar muscle mass. In particular, patient positioning (and specifically the head of the bed elevation) can significantly influence ultrasound measurements, and significant differences in RF CSA were recently reported across four different hip flexion positions (0 to 60 degrees) [96]. Moving from standing to recumbent position leads to positional fluid shifts that have been shown to influence the ultrasound measures for up to 45–60 min [97, 98]. Moreover, this affects some muscle groups and some measurements more than others, so that CSA is affected more than MT [99]. Besides the effect of fluid shift, the position per se seems to influence the ultrasound measurements: a sitting position with hips and knees at a 90° flexion induces an increase in anterior thigh thickness as compared to the supine position [100]. Eventually, some authors reported differences in size and functions of the muscles of the dominant vs. non-dominant side [101].

A small investigation revealed a certain degree of asymmetry in muscle wasting between the legs, indicating that the right leg experienced a slightly greater loss of muscle mass over time [33]. The majority of studies scanned the quadriceps muscle along a line connecting the anterior superior iliac spine (ASIS) and the patella. However, the distance between these two structures at which the measurement was taken varied greatly, being reported as two-thirds, one-half, three-fifths, 10–15 cm proximal to the patella, or a combination.

The application of pressure with the ultrasound probe has been demonstrated to flatten muscles by over 50% of their original size [102]. Given that the distortion occurs over the superficial-deep axis, resulting in a probable increase in the lateral diameter of the muscle, it is likely that this compression affects Muscle Thickness (MT) more significantly than Cross-Sectional Area (CSA). This observation might elucidate why CSA appears to be more reliable than MT [87]. In summary, it is likely that combined effect of heterogeneity of the measurement protocols and the variability of the findings among studies prevented the identification of a meaningful cut-off and calls for a standardization of the technique.

A clear cutoff for normal or reduced values of muscle mass is still lacking for the critically ill population. In different populations, however, ultrasonographic measurements have been found to be well correlated with reference methods for the assessment of muscle mass, such as CT scan and MRI-based evaluations [103]. Indeed, a recent investigation from our group found how a reduced baseline RF-CSA, as assessed by muscle ultrasound, was associated with increased ICU mortality, independently of age, the type of ICU admission, and the nutritional risk, and proposed a value of 2.39 cm [2] as the best cutoff to predict an adverse outcome; notably, the absolute amount of muscle mass, rather than its value normalized by body size, seems responsible for the association with mortality [104].

Indeed, no ideal site for the assessment of muscle mass has been identified. Notably, not all peripheral muscles decline at the same extent [105], and little consistency in terms of anatomic landmarks, probe placement, patient positioning, ultrasound settings and the amount of compression has been reported and summarized in the literature [106]. A recent study showed that, in a population of non-critically-ill subjects, age- and sex- standardization and a precise protocol for ultrasound scanning led to an excellent agreement for the measurement of muscle mass with reference standards [107]. With this regard, in the general population of non-critically ill subjects, consensus guidelines are starting to be published and disseminated on the optimal technique for each muscle site and measurement, with the aim of improving the external validity of muscle ultrasound [108]. Notably, the intra- and interclass correlation coefficient reported in the different studies included in the current review indicate good or excellent reliability, which suggests that if a specific protocol is followed, the measure is reproducible, again calling for an effort for a standardization of the procedure.

In the meta-regression, we identified how an older age and high BMI are associated with reduced muscle mass. On the one hand, it is well known how an advanced age is the main non-modifiable risk factor for malnutrition; moreover, older persons are particularly susceptible to disease-related weight loss and the loss of muscle mass and strength (i.e., sarcopenia) [109]. On the other hand, malnutrition screening tools such as the NUTRIC score or NRS-2002, or the GLIM diagnostic criteria, consider the effect of BMI only in terms of a higher risk for excessively low BMI values, while from the findings we have highlighted in the meta-regression it seems more evident the opposite effect, i.e. the association between a high body mass index and reduced muscle mass. In fact, in recent years it is increasingly emerging that sarcopenic obesity, i.e. the coexistence of a high body mass index and reduced muscle mass and function, is a more frequent occurrence than previously considered, even in critically ill patients [110], and how this condition is associated with unfavourable outcomes [111]. From a practical point of view, it might be reasonable to suggest the systematic use of ultrasound assessment of muscle mass in categories of patients at greater risk, such as the elderly and obese, who could benefit from greater attention to muscle mass monitoring given the greater risk of malnutrition and sarcopenia, in order not only to diagnose manifest malnutrition but also to capture the risk to develop malnutrition as described above, with the purpose of identifying patients early in order to titrate the nutritional treatment.

Based on the aims of the included studies, most of the papers used muscle ultrasound as a method for the assessment of body composition upon ICU admission, or for longitudinal analysis of muscle mass loss. This highlights the acknowledgement that a reduced muscularity or the loss of muscle mass represents an unfavorable condition associated with adverse events [52], and underlines the usefulness of a method for quantifying muscle mass at the bedside [14]. On the other hand, a relevant proportion of the studies included in the current review used ultrasound as a method to assess the effects of specific interventions. Various recent studies are showing how this method can indeed influence nutritional strategies: energy intake in acute patients was found to be associated with muscle mass as assessed by ultrasound [112]; similarly, protein intake was associated with changes in rectus femoris and diaphragm size [29].

Besides the current applications of muscle ultrasound, summarized in the paragraph above, some reports are pointing out further potential clinical applications. In a recent study, it was investigated whether, as suggested [113], the assessment of body composition could lead to an individualisation of protein intake. The authors pointed out that a higher protein intake was associated with lower ICU mortality only in patients admitted with a higher muscle mass, as assessed by muscle ultrasound [114].

The findings of this systematic review have to be considered in the light of the limitations of the study. In particular, a predominant portion of the included studies was observational, and in instances where the design was controlled, muscle ultrasound served primarily to evaluate the intervention’s impact rather than as a parameter guiding therapeutic strategies. Moreover, we only focused on ultrasound parameters of muscle mass, and did not include studies which investigated parameters of muscle quality or function, such as the echointensity or the pennation angle. We did not gather information about which professional figure had performed the ultrasound examination, and what his/her degree of experience was, which might have given us a hint about the professionals who use this imaging technique. Only a limited amount of data was available for the meta-regression analysis: we were not able to investigate the effect of nutritional status because of the heterogeneity of the definitions, and limited data on disease severity could be retrieved and analyzed. Additionally, the studies were frequently characterized by modest sample sizes and/or heterogeneity, thereby constraining the external validity of the findings and the significance of any aggregate analysis. Furthermore, there was a scarcity of data concerning pre-admission baseline characteristics and the functional state of patients, hindering the assessment of how these factors might influence muscle mass and loss.

In conclusion, the different studies on muscle ultrasound had high reliability and internal validity. However, external validity and generalizability were generally lacking, leading to a significant heterogeneity in the values reported. Differences in the characteristics of the subjects included, in the identification of anatomic landmarks and in the technique of image acquisition might at least in part explain such variability and suggest the need for standardization of measurements and for age- or sex-specific cutoffs. Eventually, besides the need for a higher degree of methodological standardization, future studies should consider other aspects of lower limb muscle ultrasound that were not addressed in the current review, specifically clinimetric properties such as construct validity and predictive ability, to further support its use in clinical practice.

## Electronic supplementary material

Below is the link to the electronic supplementary material.

Supplementary Material 1

## Data Availability

The datasets used and/or analysed during the current study are available from the corresponding author on reasonable request.

## Abbreviations

AIIS: Anterior inferior iliac spine
ASIS: Anterior superior iliac spine
D: Days of ICU stay
ECMO: Extra corporeal membrane oxygenation
FU: Follow-up
ICU: Intensive care unit
ICU - AW: Intensive care unit acquired weakness
Max: Maximum
Min: Minimum
NA: Not available
Pts: Patients
RFcsa: Rectus femoris cross sectional area
RFM: Rectus femoris muscle
SBP: Superior border of patella
SIC: Superior iliac crest
