# Supplementary material for: Methodologies and clinical applications of lower limb muscle ultrasound in critically ill patients: a systematic review and meta-analysis
Source: Ann Intensive Care. 2024 Oct 24;14:163. doi: 10.1186/s13613-024-01395-y (PMC11499498; doi:10.1186/s13613-024-01395-y)

**Methodologies and clinical applications of lower limb muscle ultrasound in critically ill patients: a systematic review and meta-analysis**

*Running title: Lower limb muscle ultrasound in the critically ill*

Roberto Venco*, Alessandro Artale*, Paolo Formenti, Cristian Deana, Giovanni Mistraletti,

Michele Umbrello

* These two authors contributed equally to the present manuscript

**SUPPLEMENTARY APPENDIX**

**Table s1 - Summary of the inclusion and exclusion criteria of the systematic review**

| Inclusion criteria | Exclusion criteria |
| --- | --- |
| Adult  Critically ill patients admitted to ICU  Patients who underwent muscle ultrasound monitoring of the lower limb during their ICU stay  Studies that described methods and clinical applications of muscle ultrasound of the lower extremities  Prospective or retrospective observational cohort studies  Non-randomized and randomized controlled trial  Studies with full text available in English | Case reports  Case series  Reviews  Meta-analyses  Pediatric studies  Letters to editor  Studies not in English  Studies lacking full text |

**Table s2 - Summary of the studies included in the meta-analysis: year of publication, setting, design, number of patients, inclusion and exclusion criteria, ultrasound measurement employed**

| **Author** | **Year** | **Setting** | **Design** | **N pt** | **Inclusion criteria** | **Exclusion criteria** | **Measure** |
| --- | --- | --- | --- | --- | --- | --- | --- |
| ***Gruther et al.*** | 2008 | Medical University of Vienna | Prospective observational | 17 | >19 y.o, admitted to ICU | ICU LOS < 28 days, neuromuscular disorders or critical illness neuropathy, palliative care | QMLT |
|  |  |  | Cross-sectional | 101 | >19 y.o, admitted to ICU | ICU LOS < 7 days, neuromuscular disorders or critical illness neuropathy, palliative care | QMLT |
| ***Gerovasili et al.*** | 2009 | Evangelismos Hospital, Athens, Greece | RCT | 26 | > 18 y.o, admitted to ICU | ICU LOS < 48 h, pregnancy, obesity (BMI >35 kg/m2), brain death, history of neuromuscular disease, diseases with systemic vascular involvement, technical obstacles that did not allow EMS, patients with PM | RFMT,  VIMT |
| ***Cartwright et al.*** | 2012 | Wake Forest Baptist Hospital, Winston-Salem, USA | Prospective observational | 16 | Adults, admitted to ICU, MV for ARF, no underlying neurological conditions | NA | RFMT, TAMT |
| ***Puthucheary et al.*** | 2013 | King’s College Hospital NHS Trust, Whittington Hospital NHS, London, UK | Prospective observational | 63 | >18 y.o, anticipated to be intubated > 48h, spend > 7 days in critical care, survive ICU stay | Pregnancy, lower limb amputated, history of neuromuscular pathology, disseminated cancer | RFcsa |
| ***Baldwin et al.*** | 2013 | Flinders Medical Centre, Bedford Park, Australia | Cross-sectional | 32 | ≥ 18 y.o, septic, admitted to ICU, receiving MV ≥5 days,> 2 weeks after open upper abdominal or thoracic surgery, no hemodynamic, respiratory, or mobility restriction that precluded body weight measurement, minimal ventilation support sufficient for respiratory muscle measurements, sufficient wakefulness (RASS of -1 to +1 and GCS ≥ 14, no dialysis at the time of measurements or in the previous 24h | open surgery, anticipated hospital admission <14 days, history or acute central nervous system, neuromuscular pathology, COPD, chronic heart failure (NYHA class III or IV), cystic fibrosis, admission related to self-harm, cognitive/intellectual impairments or psychiatric admission, alcohol-related admission, liver failure, or dependence, obesity, cancer or metastatic disease, history of chronic systemic inflammatory condition or current immunosuppression, prolonged hospitalization in the previous 3 months, anticipated HLOS ≤ 5 days, non–English-speaking, pregnancy, abnormal chest wall or thoracic spine anatomy, anorexia, severe burns | QMLT |
| ***Sarwal et al.*** | 2014 | Winston-Salem, USA, Austin Health, Melbourne, Australia | Cross-sectional | 20 | admitted to ICU | NA | QMLT |
| ***Parry et al.*** | 2015 | Austin Hospital, Melbourne, Australia | Prospective observational | 22 | Adults, receiving MV > 48h, ICU LOS > 4 days | Spinal cord injury, history of neuromuscular disease, new intracranial process | RFcsa, RFMT,  VIMT,  VLMT |
| ***Mueller et al.*** | 2015 | Massachusetts General Hospital and Harvard Medical School, Boston, USA | Prospective observational | 102 | >18 y.o, ICU LOS >24h | transferred from skilled nursing facility, ongoing discussions about goals of care, Motor on GCS<5, history of paralysis, pregnancy, no proxies available for consent absence of lower limbs | RFcsa |
| ***Francis et al.*** | 2016 | Royal Columbian Hospital, New Westminster, Canada | Prospective observational | 8 | admitted to ICU | history of diaphragmatic or neuromuscular disease, obesity (BMI > 40 kg/m2) | QMLT |
| ***Paris et al.*** | 2016 | University of Waterloo, Ontario, Canada | Prospective observational | 149 | cross-sectional abdominal CT scan of at least the L3 vertebra performed for clinical reasons <24h before or <72 h after ICU admission, patients with BMI <25 kg/m2 and BMI >35 kg/m2 | palliative care | QMLT |
| ***Turton et al.*** | 2016 | Royal Liverpool University Hospital, UK | Prospective observational | 22 | >18 y.o, assented within 24h of being intubated and admitted to ICU | pregnancy, trauma to either the right lower or right upper limbs, history of neuromuscular diseases, rhabdomyolysis, vascular insufficiency or amputation of the right upper or lower limbs, and prolonged immobility prior to admission | VLMT |
| ***Annetta et al.*** | 2017 | Policlinico A. Gemelli, Rome, Italy | Prospective observational | 38 | 18–59 y.o, trauma patients, ISS > 25, well-nourished, previously healthy, no history of nutritional problems, chronic use of drugs, orthopedic issues in the previous 2 years | relevant comorbidities, history of immune abnormalities (including treatment with corticosteroids), neuromuscular disease, cancer | RFcsa, RFMT, TAcsa, TAMT |
| ***Connolly et al.*** | 2017 | King’s College Hospital, London, UK | Prospective observational | 21 | ≥ 18 y.o, admitted to ICU, receiving MV > 48h, ICU LOS >7 days | acute neurological diagnoses, history of neuropathies or myopathies, receipt of neuromuscular blocking agents, trauma injuries (precluding lower limb strength assessment), pregnancy | RFcsa,  TAcsa |
| ***Chapple et al.*** | 2017 | Major acute neurotrauma referral centre for South Australia | Prospective observational | 37 | > 18 y.o, ICU for >48h, with a moderate TBI (GCS 9–12) or a severe TBI (GCS score, 3–8), | palliative care | QMLT |
| ***Palakshappa et al.*** | 2018 | Hospital of the University of Pennsylvania | Prospective observational | 29 | sepsis complicated by respiratory failure (need for MV, NIMV, HFNC with FiO2 > 50%) or shock requiring vasopressors > 6h, an anticipated ICU LOS >48h | transferred from a long-term acute care facility, outside hospital > 48 h after the onset of their critical illness, tracheostomy at admission, admitted to the ICU for at least 7 days in the prior 3 months, non-English speaker, pt cognitively impaired or unable to follow physical therapy commands, history of neuromuscular disease, or paresis of bilateral lower extremities, and acute spinal cord injury | QMLT,  RFcsa |
| ***Pardo et al.*** | 2018 | Saint-Antoine University Hospital, Paris, France | Prospective observational | 29 | > 18 y.o admitted to the ICU, ICU LOS >7 days, received a muscular US evaluation as part of their usual care | history neuromuscular pathology, lower-limb amputation, whose US data were missing or incomplete, ICU LOS < day 7 | QMLT |
| ***Katari et al.*** | 2018 | Rajarajeswari Medical College and Hospital, Bangalore, India | Prospective observational | 100 | 18–90 y.o, admitted in ICU, anticipated ICULOS > 7 days | pregnancy, amputated lower-limb, history of neuromuscular pathology, disseminated cancer, long-term critically ill patients shifted from other hospitals | QMLT, RFMT |
| ***K. Hayes et al.*** | 2018 | The Alfred Hospital, Melbourne, Victoria, Australia | Prospective observational | 25 | adults expected to be on ECMO for 24h | >48h on ECMO or 5 days in ICUprior to recruitment, any connective tissue disorders, any neuromuscular conditions, any current cancer or chemotherapy, any current acute musculoskeletal injuries of hip, knee, and ankle, a pre-existing mobility impairment where the patient was unable to walk without assistance, unable to cooperate, unable to obey to verbal command, where death was deemed imminent and inevitable, or where the US assessor was away on leave | QMLT, RFcsa, RFMT,  VIMT,  VLMT |
| ***Hernández-Socorro et al.*** | 2018 | University Hospital in Las Palmas de Gran Canaria | Prospective observational | 48 | no malnutrition prior to the admission, required prolonged mechanical ventilation, expected to stay >7 days in ICU | Patients who were not expected to survive > 3 days, history of neuromuscular pathology | RFcsa, RFMT |
| ***Woo et al.*** | 2018 | Yonsei University College of Medicine, Seoul, Korea |  | 10 | > 20 y.o, admitted to ICU, receiving MV >24h | hemodynamically unstable, unable to cooperate or to obey verbal command, and had wound or injury on the legs | RFcsa |
| ***Twose et al.*** | 2018 | University Hospital of Wales, Heath Park, Cardiff, UK | Prospective observational | 26 | ≥ 18 y.o, admitted to ICU, receiving MV, ≥6kPa, PaCO2 4.6kPa<PaCO2< 5.9kPa | Patients with palliative intention, history of neuromuscular disease, or chronic respiratory disease | RFcsa |
| ***Martin et al.*** | 2018 | Hospital San Angel Inn Universidad, Ciudad de Mexico, Mexico | Prospective observational | 59 | >18 y.o, admitted to ICU, with a minimum of 48h of stay | neuromuscular diseases, hip fracture, right femur fracture, right thigh burn, any procedure made on the right leg, right lower limb amputation, pregnancy, palliative care, transferred to other hospitals, less than 48h of stay | QMLT |
| ***Mukhopadhyay et al.*** | 2018 | National University Hospital, Singapore | Prospective observational | 48 | ≥21 y.o, critically ill patients, expected duration of MV and HLOS of at least 72h and 10 days | amputation, surgery, neurological, or rheumatological problems involving the lower limbs | RFcsa |
| ***Silva et al.*** | 2018 | University Hospital of Brasília, Brazil | Prospective observational | 22 | 18-60 y.o, TBI patients admitted to the ICU, receiving MV | pregnancy, BMI >35 kg/m2,history of neuromuscular disease, chronic renal insufficiency, autoimmune diseases, bone fractures or skin lesions at the evaluation site, uncontrolled cranial hypertension, clinical suspicion of brain death | QMLT, TAMT |
| ***Fetterplace et al.*** | 2018 | Royal Melbourne Hospital, Australia | RCT | 60 | >18 y.o, MV for <48h, and were anticipated to remain ventilated for at least 72h | contraindication to enteral feeding, palliative care, premorbid disability resulting in an inability to ambulate >10 m, pregnancy, or the treating clinician considered the intervention was not in the patient’s best interest | QMLT |
| ***Borges et al.*** | 2019 | University Hospital, Sao Paulo, Brazil | Prospective observational | 37 | >18 y.o, admitted to ICU, severe sepsis or septic shock within 24h of evolution in another unit | previous or current history of stroke with physical limitation, neuromuscular diseases, other types of associated shock, polytraumatized limb fracture, palliative care, ICU LOS < 72h, not obtained informed consent term | RFcsa |
| ***Borges et al.*** | 2019 | University Hospital, Sao Paulo, Brazil | Prospective observational | 45 | >18 y.o, admitted to ICU, severe sepsis or septic shock within 24h of evolution in another unit | previous or current history of stroke with physical limitation, neuromuscular diseases, other types of associated shock, polytraumatized limb fracture, palliative care, ICU LOS < 72h, not obtained informed consent term | RFcsa |
| ***Silva et al.*** | 2019 | Hospital de Base do Distrito Federal, Brasília, Brazil | RCT | 60 | 18 -60 y.o, MV for up to 24h, severe TBI | history of alcoholism, HIV, chronic kidney failure, spinal cord injury, pregnancy, skin lesions in the region to be treated, unstable fractures in the vertebral column and lower limbs | RFMT, TAMT |
| ***Özdemir et al.*** | 2019 | Gazi University Faculty of Medicine, Ankara, Turkey | Prospective observational | 55 | >18 y.o, admitted to ICU | muscular atrophy in lower extremities due to cerebrovascular accident, neuromuscular disease, or trauma, ICU LOS < 48h | QMLT |
| ***Nickels et al.*** | 2020 | Princess Alexandra Hospital, Brisbane, Australia | RCT | 72 | > 18 y.o, expected to be MV > 48h, recruited <96h of ICU admission, expected ICU LOS >48h from study enrolment | history of impaired mobility, new neurological disorder, injuries precluding in-bed cycling,> 135 kg, pregnancy, uncontrolled seizures or status epilepticus, palliative care | RFcsa, RFMT,  VIMT |
| ***Pita et al.*** | 2020 | Keck Hospital of University of South California, USA | Prospective observational | 50 | >18 y.o, who underwent evaluation for potential listing or were listed for Liver Transplantation, admitted to CU | NA | RFcsa |
| ***Mayer et al.*** | 2020 | University of Kentucky College of Health Sciences, Lexington, USA | Prospective observational | 41 | ≥ 18 y.o, admitted to ICU, diagnosis of ARF or sepsis, anticipated to spend more than 3 days in the ICU and survive the current hospitalization, enrolled within 48h of admission | cognitive impairments, non-ambulatory prior to hospitalization, history of neuromuscular disorder, new traumatic injury with lower-extremity fracture, one or more amputations of lower-extremity, pregnancy, admitted for substance abuse, BMI > 45 kg/m2, otherwise inappropriate for study procedures as determined by the primary attending physician | QMLT, RFcsa, RFMT, TAcsa, TAMT |
| ***Fetterplace et al.*** | 2020 | Royal Melbourne Hospital, Australia | Retrospective observational | 35 | abdominal CT scan within 72 h of one of the QMLT measurements | CT scan images inappropriate for analysis owing to the lack of clarity and artifacts or if the patient's positioning meant that the skeletal muscle was incompletely visualized | QMLT |
| ***Z.-Y. Lee et al.*** | 2020 | University of Malaya, Kuala Lumpur, Malaysia | Prospective observational | 86 | >18 y.o, receiving MV within 48h of ICU admission and expected to stay in the ICU for at least 96h | Pregnancy, palliative care, amputated or injured lower limbs and bilateral deep vein thrombosis. patients who stayed in the hospital >5 days in the past 2 weeks and not ambulating independently prior to illness that led to this ICU admission | QMLT,  RFcsa |
| ***Dimopoulos et al.*** | 2020 | National and Kapodistrian University of Athens, Athens | Prospective observational | 165 | >18 y.o, following admission in the Cardiac Surgery ICU within 24 h of cardiac surgery | unable to get a muscle US assessment within 24 h of admission in the ICU, obesity (BMI) > 35kg/m2, patients with open chest-sternotomy, lobectomy or ECMO, extensive peripheral thigh edema and preexisting neuromuscular disease, patients who were re-admitted to the ICU | QMLT,  RFMT |
| ***Tourel et al.*** | 2020 | Centre Hospitalier Universitaire de Saint-Etienne, Univ Lyon | Prospective observational | 38 | GCS ≤ 8 at admission, receiving MV, and if patients were a priori scheduled for iterative follow-up brain CT scans. A maximum of 3 CTs was used in the study during the 10 first days of admission | bilateral leg injury, obesity (BMI > 35 kg/m2) | QMLT |
| ***McNelly et al.*** | 2020 | Royal London Hospital, UK | RCT | 121 | > 18 y.o, expected to be intubated and ventilated for > 48h, requiring enteral nutrition via nasogastric tube, SOFA score > 2 in > 2 domains at admission, likely ICU stay >7 days and likely survival > 10 days (assessed as previously by senior ICU clinicians). | pregnancy, unilateral/bilateral lower limb amputation, recruitment into trials of interventions affecting muscle mass, preceding enteral feeding during this hospital admission and >12h on ICU, unlikely to meet nutritional requirements by 72 h using a standard feeding schedule, ICU admission within the previous 3 months, need for sole/supplemental parenteral nutrition or post-pyloric feeding, ECMO, diagnosis of active disseminated malignancy or primary neuromyopathy | RFcsa |
| ***Nakanishi et al.*** | 2020 | Tokushima University Hospital, Japan | RCT | 36 | > 18 y.o, expected receiving MV> 48h and to stay in the ICU > 5 days, recruited within 24 h following ICU admission on weekdays. | trauma or amputation of upper and lower limbs, history neuromuscular disease, SBP< 80 mm Hg even with inotropic or vasopressor support, HR<40 or >140 beats/min, and peripheral oxygen saturation less than 88% with ventilatory support. died before day 5, withdrawn due to pain | QMLT,  RFcsa |
| ***Nakanishi et al.*** | 2020 | Tokushima University Hospital, Japan | Prospective observational | 56 | > 18 y.o, ICU LOS > 5 days | surgery not including percutaneous abscess drainage, chest tube insertion, and tracheostomy in ICU, pregnancy, diagnosis of primary neuromuscular disease, trauma at the measurement point, and unclear US image | RFcsa |
| ***YongpengXie et al.*** | 2020 | Hospital of Lianyungang City, China | Prospective observational | 95 | >18 y.o, conscious patients, receiving MV >5 days, ICU LOS ≥7 days | spinal cord injury, acute stroke, lower limb fracture, history of cognitive dysfunction or neuromuscular disease, receiving muscle relaxant treatment, acute and chronic heart failure, severe edema, Patients with HLOS less than 7 days due to death or automatic discharge | RFcsa |
| ***Dresen et al.*** | 2021 | University Hospital of Bonn, Germany | RCT | 42 | >18 y.o, MV, overcoming the early period of hemodynamic instability according to the ESPEN’s, prediction of a long-term ICU stay (> 28 days) | terminal chronic renal failure, persistent ARDS, ECMO, history myopathies, traumatic brain injuries, intracerebral hemorrhages, cerebral ischemia followed by muscular failures, inhalation sedation | QMLT |
| ***Yanagi et al.*** | 2021 | Kitasato University Hospital, Sagamihara, Japan | Prospective observational | 72 | ICU admission, predicted ICU stay to stay ≥ 48h | Patients who could not independently perform basic ADLs, died in ICU | QMLT |
| ***Viana et al.*** | 2021 | Lausanne University Hospital, Switzerland | RCT | 30 | >18 y.o, MV, CVC, functional gastrointestinal tract, receiving a full treatment, assessed likely to survive > 7 days | major burns (>20% BS), admission for cardiorespiratory arrest or brain injury, pregnancy or lactation, parenteral nutrition, participation in another interventional trial, absence of consent | QMcsa |
| ***Supinski et al.*** | 2021 | University of Kentucky, Lexington, USA | RCT | 73 | adult, receiving MV > 48h for respiratory failure | unstable patients to tolerate measurements, received neuromuscular blocking agents < 48h preceding testing, history neuromuscular disease, recent variceal bleeding, pregnant, incarcerated, institutionalized, palliative care | QMLT |
| ***Baston et al.*** | 2021 | University of Washington, USA | Prospective observational | 15 | > 18 y.o, admitted to the ICU | pregnancy, prisoner, deemed medically unstable, open wounds or burns at US study sites, imminent death expected | QMLT, RFcsa, TAMT |
| ***Umbrello et al.*** | 2021 | Ospedale San Carlo Borromeo, Milan, Italy | Prospective observational | 28 | > 18 y.o, admission for acute hypoxemic respiratory failure, MV > 48h, SARS-CoV-2 infection | pregnancy, trauma to the right lower limb, history of neurologic, neuromuscular or muscular wasting disease, prolonged immobility before ICU admission | RFcsa |
| ***Bury et al.*** | 2021 | Cleveland Clinic Hospital, Ohio, USA | Prospective observational | 52 | > 18 y.o, admitted to the SICU <72 h prior to evaluation, expected to remain in the ICU, fed solely via nutrition support for at least 3 consecutive days | ICU LOS >72 h prior to study enrollment, HLOS > 5 days prior to study enrollment, Anticipated ICU LOS ≤3 days after study enrollment, NS not expected to be sole source of nutrition during study period, Boarding from another ICU, Pregnancy: Comfort care/hospice, Oral diet greater than clear liquids, On protein modular, Received solid organ transplant during current admission,Admitted directly from skilled nursing or long-term acute care facility, Trauma to both lower extremities | QMLT |
| ***Lambell et al.*** | 2021 | Alfred Hospital, Melbourne, Australia | Prospective observational | 50 | ≥18 y.o, a CT scan including the L3 area performed for clinical purposes ≤24 h before or ≤72 h after ICU admission | unanalyzable CT scan, imminent death, anticipated ICU stay of <24 h, pregnancy, impracticality and/or incapability to perform theUS protocol or to obtain consent, BMI > 40 kg/m2 | QMLT |
| ***Rodrigues et al.*** | 2021 | Hospital das Clínicas da UFMG, Belo Horizonte, Brasil | Prospective observational | 60 | ≥18 y.o, admitted to the ICU, expected to stay for >72 h in ICU | Pregnancy and puerperal women, re-hospitalized patients, previous history of lower-limb paresis | QMLT,  RFcsa |
| ***Er et al.*** | 2021 | Hacettepe University Faculty of Medicine, Ankara, Turkey | Prospective observational | 38 | > 18 y.o, enrolled in the first 36 h of intubation | died in the first day of ICU, no attempt for weaning, IMV >48h in the last 6 months, refused to give consent, US could not be performed within the first 36h | QMLT, RFMT |
| ***Tanaka et al.*** | 2021 | Osaka police Hospital, Japan | Prospective observational | 8 | septic shock, expected to require MV for at least 1 week | Cardiopulmonary arrest, palliative care, expected to receive extubation within a few days, ultrasonography that could not be carried out | RFMT |
| ***Toledo et al.*** | 2021 | Hospital das Clínicas, Universidade de Sao Paulo, Brazil | Prospective observational | 74 | ≥ 18 y.o, admitted to the ICU, with expecting to need mechanical ventilation for at least 48h | history neuromuscular diseases, any amputated lower limbs, underwent orthopedic surgery of the lower limb, need home ventilation, in the prone position, transferred from another hospital after staying >48h, refused to provide consent, patients with extubating<48h, HLOS <48h | QMLT |
| ***Zhang et al.*** | 2021 | RuiJin Hospital, Shanghai, China | Prospective observational | 37 | ≥ 18 y.o, anticipated ICU stay of at least 2 days | generalized or regional weakness or with any diagnosis at the time of admission, unable to follow commands, edema of upper and lower limbs, amputated arms or legs, wounds, fractures, lesions, burns, or bleeding at the measurement points, patients who received early mobilization or physical therapy during the observation period | RFcsa, RFMT,  VIMT |
| ***Arai et al.*** | 2021 | Tokushima University Hospital and Tokushima Prefectural Central Hospital, Japan | Retrospective observational | 89 | ≥ 18 y.o, admitted to ICU, expected to stay in ICU for >5 days, underwent the US assessments of the rectus femoris muscle at the day of ICU admission, underwent the CT assessments of the L3 within 2 days before and after ICU admission | primary neuromuscular disease, obstacles at the US measurement site | QMLT,  RFcsa |
| ***Hernández-Socorro et al.*** | 2021 | Hospital Universitario de Gran Canaria, Spain | Prospective observational | 43 | patients were not malnourished prior to ICU admission, needed prolonged MV(>14 days) and expected ICU LOS > 7 days | NA | RFcsa, RFMT |
| ***L.B. da Silva Passos et al.*** | 2021 | Clinical University Hospital, Federal University of Uberlandia, MG, Brazil | Prospective observational | 160 | >18 y.o, critically ill patients with a HLOS > 72h and an expected HLOS > 48h, receiving MV or were intubated within 48h after ICU admission | Patients contraindicated for the BIA exam, limb amputation, pregnancy and lactating women, brain death since ICU admission | RFcsa |
| ***Mendes et al.*** | 2022 | Cardiology Hospital, Recife, Brazil | Prospective observational | 78 | adults, admitted in the ICU for acute cardiovascular disease | history of paralysis, lower limb amputation, coming from other ICUs, in the postoperative period, LOS < 7 days | RFMT |
| ***Formenti et al.*** | 2022 | San Paolo Hospital, Milan, Italy | Prospective observational | 32 | >18 y.o, intubated, SARS COV 2 infection, ARDS, sedated and paralyzed | history of severe COPD, pregnancy, failure to perform respiratory muscle US | RFcsa, RFMT |
| ***Maskos et al.*** | 2022 | University Hospital, Munich, Germany | Prospective observational | 40 | > 18 y.o, admitted to the NICU, neurologic etiology for ICU admission, expected LOS > 7 days | Bifrontal craniectomy, confinement to bed prior to ICU admission, recent hospitalization, renal replacement therapy, other primary neuromuscular disease, disseminated cancer, pregnancy | RFMT |
| ***Anand et al.*** | 2022 | All India Institute of Medical Sciences, New Delhi, India | Prospective observational | 48 | > 18 y.o, admitted to ICU, diagnosis of sepsis, expected to have ICU LOS >7 days | failure to obtain informed consent, paraparesis, quadriparesis, primary systemic neuromuscular disease, deep venous thrombosis | QMLT |
| ***Formenti et al.*** | 2022 | Ospedale San Paolo, Milan, Italy | Prospective observational | 96 | ≥18 y.o, MV within 24h after admission to ICU, expected ICU LOS ≥48h | end-stage kidney disease undergoing long-term dialysis, pregnancy, patients with PM or ICD, preexisting neuromuscular disease | RFcsa |
| ***Formenti et al.*** | 2022 | Ospedale San Paolo, Milan, Italy | Prospective observational | 49 | > 18 y.o, MV, expected ICU LOS ≥72h | preexisting neurological weakness, pregnancy, malignancy, routine overnight postoperative surgical recovery | RFcsa |
| ***Kangalgil et al.*** | 2022 | Karadeniz Technical University, Trabzon, Turkey | Prospective observational | 35 | 18 - 79 y.o, trauma and major surgical patients, expected ICU LOS >7 days | palliative care, pregnancy and puerperal women, obesity (BMI >40), history of neurological stroke with physical limitation, primary neuromuscular disease, diseases with systemic vascular involvement, end-stage malignancy, kidney failure on dialysis dependency, transplantation, spinal cord injury, a lower limb that was amputated, | RFcsa |
| ***Wu et al.*** | 2022 | Taoyuan General Hospital, Taiwan | Prospective observational | 284 | patients admitted to the SICU | wound on the right thigh | RFMT,  VIMT |
| ***Chapple et al.*** | 2022 | Royal Adelaide Hospital, Australia | RCT | 80 | ≥ 18 y.o, receiving MV, about to commence EN or EN had commenced within the previous 12h, expected receive EN in the ICU beyond the calendar day after randomization | informed consent was unable to be obtained, the first US measure was unable to be conducted within 48h of randomization | QMLT |
| ***Hrdy et al.*** | 2023 | University Hospital, Jihlavska, Brno, Czech Republic | Prospective observational | 104 | ≥ 18 y.o, the physician’s subjective evaluation that the patient would require MV > 48h | Clinical Frailty Score > 7 prior to admission, history of neuromuscular disease, amputated lower extremities, prior trauma to the lower extremities involving thighs and inability to cooperate with US examinations | RFcsa |

Abbreviations: ARF: acute respiratory failure; BMI: body mass index; CT: computed tomography; EMS: electrical muscle stimulation; EN: enteral nutrition; GCS: Glasgow Coma Scale; HFNC: high flow nasal canula; HLOS: Hospital length of stay; ICD: implantable cardioverter defibrillator; ICU: Intensive Care Unit; ISS: injury severity score; LOS: length of stay; MV: mechanical ventilation; NIMV: non invasive mechanical ventilation; NICU: Neurological Intensive Care Unit; PM: pacemaker; QMLT: quadriceps muscle layer thickness; QMcsa: quadriceps muscle cross sectional area; RASS: Richmond Agitation-Sedation Scale; RFcsa: rectus femoris cross sectional area; RFMT: rectus femoris muscle thickness; RCT: randomized controlled trial; SICU: Surgical Intensive Care Unit; TAMT: tibialis anterioris muscle thickness; TAcsa: tibialis anterioris cross sectional area; TBI: traumatic brain injury; US: ultrasound; VIcsa: vastus intermedius cross sectional area; VIMT: vastus intermedius muscle thickness; VLcsa: vastus lateralis cross sectional area; VLMT: vastus larteralis muscle thickness; y.o: years old.

**Table s3 – Aim of the study and Main Findings for each included study**

| **Author** | **Aim of the study** | **Measure** | **Main Findings** |
| --- | --- | --- | --- |
| ***Gruther et al.*** | To measure muscle wasting in patients in the ICU,  to determine whether a relationship could be identified betweena representative population of patients in the ICU and their ICU LOS,  to verify whether US measurements are a valid to document muscle mass. | QMLT | Muscle wasting in QMLT showed a high significant correlation with the ICU LOS.  Multiple regression analysis showed that ICU LOS was the only variable with an influence on QMLT. |
| ***Gerovasili et al.*** | To assess the effect of EMS on muscle mass preservation in critically ill patients with the use of ultrasonography. | RFMT, VIMT | EMS of lower extremities seems to preserve the muscle mass of critically ill patients as assessed with US. |
| ***Cartwright et al.*** | To evaluate whether changes occurred during ICU-stay in muscle thickness and echotexture. | RFMT, TAMT | TA and RF showed echotexture changes over 14 days: increase in mean grayscale value and a decrease in gray-scale standard deviation |
| ***Puthucheary et al.*** | To evaluateacute muscle loss in ICU patients | RFcsa | Among critically ill patients muscle wasting occurred early and rapidly during the first week of critical illness and was more severe among those with multiorgan failure. |
| ***Baldwin et al.*** | To determine muscle strength and size in the respiratory and limb muscles of ICU survivors of sepsis as compared with healthy controls, | QMLT | Critically ill patients with sepsis who require invasive MV may develop a combined generalized myopathy and ventilator-induced diaphragm dysfunction that may be recognized as a failure to wean or as ICU-acquired weakness. |
| ***Sarwal et al.*** | To determine the interobserver reliability of quantitative US measurement analyses between assessors of different expertise levels and using different techniques for selectingthe region of interest. | QMLT | Excellent interobserver reliability was obtained for all measurement techniques regardless of expertise level.  Based on these findings, it is recommended that echogenicity analysis be performed using the square technique for the quadriceps. |
| ***Parry et al.*** | To determine the rate of muscle wasting of the quadriceps muscles in ICU patients during the first 10 days from admission,  to determine the relationship between muscle US parameters and measures of muscle strength and function at ICU awakening and ICU discharge. | RFcsa, RFMT, VIMT, VLMT | Muscle wasting occurs early and rapidly within the first 10 days of admission despite usual care rehabilitation. Muscle echogenicity increased (worsened) for RF and VI muscles, by day 10.  Individuals with greater echogenicity changes had poorer function and strength. |
| ***Mueller et al.*** | To compare sarcopenia and frailty for outcome prediction in surgical intensive care unit (SICU) patients. | RFcsa | Sarcopenia defined by US of the RF muscle predicted adverse discharge disposition and hospital LOS, and is associated with higher need for institutional care after discharge, representing higher disease burden, and functional dependence after hospitalization. |
| ***Francis et al.*** | To determiner inter-operator and inter-observer reproducibility and reliability at obtaining daily US images. | QMLT | Use of ultrasound to measure QMLT was feasible and yieldedreproducible results.Thinning of the quadriceps indicates that muscle decline in critically ill patients undergoing MV was not limited to the diaphragm. |
| ***Paris et al.*** | To evaluate the validity and reliability of an US protocol that can be used to estimate muscle mass in ICU patients. | QMLT | QMLT alone may not accurately identify patients with low muscle mass. |
| ***Turton et al.*** | To establish changes in pennation angle and fascicle length as markers of altered fasciclearrangement in critical illness,  to compare muscle wasting in areas of the upper and the lower limb in ventilated patients. | VLMT | Muscle architecture in the lower, but not in the upper limb is altered within the first five days of being intubated and mechanically ventilated, potentially reducing force generation and contributing to ICU acquired weakness. |
| ***Annetta et al.*** | To evaluate the feasibility of detecting morphological muscle changes by ultrasonography over the ICU stay. | RFcsa, RFMT, TAcsa, TAMT | US is an easy, effective and practical tool for the daily estimate of changes in skeletal muscles: quantitative muscle loss is associated with an increased echogenicity; extensor muscles (RF) are much more affected than flexor muscles (TA). |
| ***Connolly et al.*** | To measure the degree and trajectory of change in peripheral skeletal muscle strength in ICU patients. | RFcsa, TAcsa | Reduced force-generating capacity was evident even on day 1 of ICU admission. No further decrements in ADMF were observed after 1 week despite the presence of significant reductions in TAcsa. Use of US offers a clinically useful tool for monitoring parameters of muscle architecture, these data fail to provide a true physiological assessment of the functional status of the muscle. |
| ***Chapple et al.*** | To describe longitudinalchanges in anthropometrical data,  to compare the accuracy of non-invasive methodologies to the validated dual-energy x-rayabsorptiometry (DXA),  to assess the relationships betweenanthropometrical data and self-reported physical function. | QMLT | A reduction in QMLT occurred in the ICU but stabilized after ICU discharge. DXA-derived total lean mass taken within 7 days of ICU discharge strongly correlated with US-derived QMLT taken within 5 days of DXA measurements.  US-derived QMLT is related to total lean mass and physical function after discharge. |
| ***Palakshappa et al.*** | To describe the relationship RFcsa and QMLT, with volitional measures of strength and function in critically ill patients. | QMLT, RFcsa | The rate of change in RFcsa over 7 days moderately correlated with strength one week after sepsis admission and may complement volitional measures of strength or function in describing skeletal muscle dysfunction in sepsis. Change in RFcsa has the stronger relationship with volitional outcome measures compared to static measures of area at admission. |
| ***Pardo et al.*** | To assess the intra- and inter-observer reliability of QMLT measured with US in ICUpatients,  to describe the QMLT evolution over the three first ICU weeks after admission and its association with nutritional intake. | QMLT | The assessment by US of the QMLT reveals good intra- and inter-observer reliability and constitutes an accurate bedside method to diagnose and monitor acute muscle wasting in critically ill patients. The “two-thirds” site showed better accuracy than the “midpoint site”. No significant relationship was found with nutritional intake. |
| ***Katari et al.*** | To measure muscle wasting and fat loss in patients in the ICU over a period of 7 days,  to verify whether US measurements are a valid and practicaltool to document muscle mass and fat thickness. | QMLT, RFMT | The RFMT and QMLT show statistically significant difference on day 3, day 7 compared to day 1. Our study demonstrates the potential utility of US for early detection and probable corrective measures to prevent ICUAW and thus hasten weaning and decrease the ICU length of stay. |
| ***Hayes et al.*** | To quantify the change in quadriceps size and echogenicity from baseline to day 10 using US in patients requiring ECMO,  to determine the relationship between US measures, muscle strength and highest mobility level. | QMLT, RFcsa  RFMT, VIMT,  VLMT | There was a significant reduction in RFcsa by day 10. Echogenicity did not change over time. There was a negative correlation between echogenicity and MRC at day 10 and HHD at day 20. At day 20, there was a moderate correlation between total muscle thickness and IMS and MRC. In patients requiring ECMO there was marked wasting of the quadriceps over the first 10 days. Ultrasound measures were related to muscle strength and highest mobility level. |
| ***Hernández-Socorro et al.*** | To analyze a novel US muscle assessment protocol in long-stay catabolicpatients versus healthy controls. | RFcsa, RFMT | Among the US quantitative changes, muscle area and thickness significantly decreased. |
| ***Woo et al.*** | To investigate if FESand in-bed cycling have positive effects on muscle massin patients receiving MV. | RFcsa | In-bed cycling increased RFcsa. Adding FES did not show differences |
| ***Twose et al.*** | To investigate the effect of hypercapnia on changes in respiratory and peripheral skeletal muscle in ICU patients receiving MV. | RFcsa | Hypercapnia did not appear to influence respiratory and peripheral skeletal cross-sectional area. |
| ***Martin et al.*** | To explore the relation between initial muscle mass and mortality in ICU adult patients. | QMLT | Patients who survived showed greater muscle thickness compared to the non survivors group, at admission. Muscle mass at admission is an independent protective factor for mortality despite severity according to the SOFA score, which showed to be a risk factor. |
| ***Mukhopadhyay et al.*** | To assess if modified Mnutric score obtained at admission to the ICU would identify patients at risk of greater muscle loss. | RFcsa | High nutritional risk patients identified by mNUTRIC score on the day of admission lost significantly more muscle than those at low risk. |
| ***Silva et al.*** | To detect whether the muscular changes in TBI patients receiving MV are only associated with disuse or additionally to neuromuscular electrophysiological disorders (NED). | QMLT, TAMT | Patientsreceiving MV, with TBI developed NED in addition to changes inmuscle structure during their stay in the ICU. TA and RF showed a significant atrophy and decrease in muscle quality. |
| ***Fetterplace et al.*** | To determine whether a highprotein volume-based enteral feeding protocol with additional protein supplementation delivered more protein andenergy than a standard hourly-rate-based nutrition protocol without protein supplementation to mechanically ventilated critically ill patients. | QMLT | A highprotein volume-based protocol with protein supplementation delivered greater amounts of protein and energy. This interventionwas associated with attenuation of QMLT loss and reduced prevalence of malnutrition at ICU discharge. |
| ***Borges et al.*** | To evaluate the association between RFcsa obtained by US and muscle strength in septic patients,  to quantify the modifications of RFcsa during the hospital stay,  to determine clinical factors associated with muscle degradation and changes in muscle strength. | RFcsa | Septic patients had a significant reduction of RFcsa during ICU stay. There was an association of RFcsa with clinical assessments of peripheral muscle strength. The organ damage score (SOFA) seems to have an independent association with muscle degradation and muscle weakness in sepsis survivors. |
| ***Borges et al.*** | To investigate the relationship between systemic inflammation and muscle changes observed by US in septic patients. | RFcsa | In septic patients, there is an association between inflammation and changes in muscle mass and strength during ICU stay. Despite a recovery of muscular strength during hospitalization, muscle mass did not present this behavior, demonstrating a significant decline in a short period of ICU stay. |
| ***Silva et al.*** | To assess the time needed and effects of NEMS on muscle architecture, NED and muscle strength. | RFMT, TAMT | NEMS applied daily for fourteen consecutive days reduced muscle atrophy, the incidence of NED, and muscle weakness in critically ill TBI patients. At least 7 days of NMES were required to elicit the first significant results. |
| ***Özdemir et al.*** | To investigate the role of the maximum compressed (MC) and uncompressed (UC) QMLT, measured byUS in the detection of nutritional risk in ICU patients with different volume status. | QMLT | US measurement of total mcQMLT can be used as a novel nutritional risk assessment parameter in ICU patients with different volume statuses. |
| ***Nickels et al.*** | To investigate if in-bed cycling in patients expected to require more than 48 h of MV was effective in reducing muscle atrophy. | RFcsa, RFMT,  VIMT | In-bed cycling did not reduce acute muscle wasting in critically ill adults. |
| ***Pita et al.*** | To assess the feasibility of bedside, serial US measurements for the evaluation muscle loss in critically ill ESLD patients,  to determine how muscle loss is associated with survival to ICU discharge and overall survival. | RFcsa | A progressive decline in RFcsa. Serial US measurements can offer real-time assessment of muscle mass loss over time and can be used to assess response to interventions aimed to ameliorate the progression of sarcopenia. Decreased RFcsa-BSA isassociated with poor overall survival. |
| ***Mayer et al.*** | To determine whether muscle alterations assessed during an ICU stay by changes in muscle size, quality, strengthand power, are associated with or predict diagnosis of ICUAW and physical function at hospital discharge. | QMLT, RFcsa, RFMT, TAcsa, TAMT | This study confirms the rapid and significant deterioration in skeletal muscle size and quality in patients admitted to the ICU for critical illness. Changes in muscle quality and power assessed in the ICU are significantly related to physical function: muscle US parameters and lower extremity muscle power measured in the ICU are significant predictors of physical function at hospital discharge. |
| ***Fetterplace et al.*** | To explore the relationship between the skeletal muscle CSA assessed using CT imaging and QMLT measurements using US with maximal and minimal pressure | QMLT | QMLT measured using US was a strong independent predictor of the mean axial skeletal muscle CSA of critically ill patients; the maximal pressure US technique appeared to perform better than the minimal pressure technique in predicting skeletal muscle CSA. |
| ***Lee et al.*** | To determine the association between baseline and changing US quadriceps muscle status with premorbid functional status and 60-day mortality. | QMLT, RFcsa | Every 1% loss of QMLT over the first week of critical illness was associated with 5% higher odds of 60-day mortality. SARC-F, CFS and mNUTRIC are associated with quadriceps muscle status and 60-day mortality and may serve as a potential simple and indirect measures of premorbid muscle status at ICU admission. |
| ***Dimopoulos et al.*** | To investigate the clinical value of skeletal muscle mass assessed by US early after cardiac surgery in terms of duration of MV and ICU LOS. | QMLT, RFMT | We reported a low rate of muscle mass loss in our study without statistical significance maybe because cardiac surgery ICU patients mostly have a short ICU stay with less severity scores as shown from our study, making the ICUAW syndrome less pronounced. |
| ***Tourel et al.*** | To validate US set-up in critically ill neurological patients,  to measure the level of agreement between measures of thigh muscle thickness derived from US and CT imaging and the intra- and inter-observer reproducibility of US measurements. | QMLT | In critically ill neurological patients at risk of developing ICUAW, a protocol of US QMLT measurement ensuring no compression during measurements has an excellent intra-rater (ICC = 0.98) and inter-rater (ICC = 0.96) reliability. |
| ***McNelly et al.*** | To study the benefits of Intermittent Feeding in the critically ill, as this may offer a more efficacious form of acute nutrition support and decrease the development of disability. | RFcsa | Intermittent feeding in early critical illness is not shown to preserve muscle mass in this trial despite resulting in a greater achievement of nutritional targets than continuous feeding. |
| ***Nakanishi et al.*** | To investigate whether EMS prevents upper and lower limb muscle atrophy and improves physical function. | QMLT, RFcsa | We found that early application of EMS prevented upper and lower limb muscle atrophy in critical ill patients. EMS also attenuated proteolysis and decreased the length of hospitalization. |
| ***Nakanishi et al.*** | To investigate the relationship between urinary titin level and limb muscle atrophy in nonsurgical critically ill patients. | RFcsa | In nonsurgical critically ill patients, urinary titin level increased 10–30 times compared with the normal level. The increased urinary titin level reflected lower limb muscle atrophy. |
| ***Xie et al.*** | To clarify the connection between GDF-15 and muscle wasting in ICU patients receiving MV, its utility as an early biomarker of muscle loss in the diagnosis of ICU-AW. | RFcsa | RFcsa loss, and % decrease in RFcsa on day 7 were found to have predictive value for ICU-AW diagnosis in mechanically ventilated patients. |
| ***Dresen et al.*** | To evaluate if a 50 % increase in daily protein supply in the later phase of critical illness could help to preserve or regain muscle mass during a long-term stay in the ICU. | QMLT | QMLT decrease over time; Nutrition intervention did not affect changes in QMLT throughout the study period. |
| ***Yanagi et al.*** | To investigate whether the assessment of sarcopenia in the ICU could help identify patients at high risk of 1-year mortality among survivors of critical illness. | QMLT | Sarcopenia in ICU, defined by low MT and low MS in the ICU, was significantly associated with 1-year mortality. The combination of skeletal muscle mass and function assessment significantly improved the accuracy of the predictive prognostic capability for 1-year mortality. |
| ***Viana et al.*** | To investigate the impact of daily pure HMB supplementation on muscle loss in critical illness. | QMcsa | 10 days of HMB did not attenuate the loss of muscle mass in a significant way within the first 15 days of the ICU stay. |
| ***Supinski et al.*** | To determine whether HMB and/or EPA improved diaphragm or quadriceps muscle strength, and/or increased their thickness, and/or reduced the time required for MV weaning. | QMLT | HMB and EPA did not improve skeletal muscle strength in medical ICU patients when given as a 10-day course. |
| ***Baston et al.*** | To conduct a standardized protocol for measuring muscle linear depth and csa in critically ill populations with a high degree of interrater agreement and feasibility. | QMLT, RFcsa, TAMT | Using a standardized protocol, US measurement of muscle in critically ill patients demonstrates good to excellent feasibility and high levels of interrater reliability. |
| ***Umbrello et al.*** | To compare the change over the first week of ICU stay in the size and quality of both RF and diaphragm muscles between critically ill, COVID-19 survivors and non-survivors,  to explore the correlation between the change in muscles size and quality with the amount of nutritional support delivered and the cumulative fluid balance. | RFcsa | Early changes in muscle size and quality may potentially be related to the outcome of critically ill COVID-19 patients and be influenced by nutritional and fluid management strategies. RFcsa was significantly reduced / RF echodensity increased after 1 week of ICU stay and this phenomenon was significantly higher in ICU non-survivors; decrease in RFcsa was significantly related to the protein deficit over the first week; increase in echodensity related to the cumulative fluid balance over the first week. |
| ***Bury et al.*** | To determine the rate of lean body mass loss in SICU patients using bedside US compared with that of age-, gender-, and body mass index (BMI)–matched healthy controls (HCs),  to correlate energy and protein delivery with the rate of muscle loss. | QMLT | Critically ill surgical patients demonstrate acute muscle loss indicated by QMLT percent change measured by bedside US that was not reflected by current recommended nutrition assessment tools. Muscle loss continues despite patients having received nearly the estimated goal energy and protein requirements by NS. |
| ***Lambell et al.*** | To compare US-derived muscle thickness at 5 different anatomical landmarks, with muscularity assessed by CT in ICU patients on admission. | QMLT | US has the potential to assess muscularity and to identify patients with low muscle mass on ICU admission. There is a strong relationship between muscularity assessed with a widely available and applicable ultrasound method and a reference method. |
| ***Rodrigues et al.*** | To evaluate the use of US as an instrument to assess the quadriceps muscle in ICU patients,comparing US-assessed muscle mass to other nutrition tools. | QMLT, RFcsa | Not observed a statistical difference in the loss of US muscle mass measurements between nourished and malnourished. |
| ***Er et al.*** | To determine the association of lower limb muscles and diaphragm thickness assessed by US within 36h of MV with weaning. | QMLT, RFMT | QMLT lower than 2.1 cm measured by ultrasound within 36 h of MV was associated with weaning failure after adjusting for BMI and Clinical frailty; increased ICU and hospital mortality. |
| ***Tanaka et al.*** | To examine changes in skeletal muscle thickness of patients admitted to the ICU for septic shock and the relationship between skeletal muscle thickness and physical function following ICU. | RFMT | Skeletal muscle thickness of septic shock patients rapidly declined after admission to the ICU in a linear manner. The rate of decrease in RFMT was correlated with ICU LOS. There appeared to be some relation between thickness of RFMT at admission and physical functioning at the 30th hospital day. |
| ***Toledo et al.*** | To determine muscle wasting assessed using US in ICU patients for 7 days and the relationship between loss of QMLT, time of MV and probability of hospital survival in critically ill patients. | QMLT | In critically ill patients with long-term of MV, QMLT quantified with US significantly decreased during the acute phase. Patients with greater muscle loss had longer time on MV. The loss of muscle thickness was significantly greater among ICU and hospital non-survive patients.The analysis techniques used by compression and non-compression ultrasonography of the quadriceps muscle were strongly correlated. A cut-off point of 1.64 cm to be a best value that discriminates loss of muscle mass when measuring by QMLT US. |
| ***Zhang et al.*** | To test the diagnostic accuracy of the changes of muscle US over time in differentiating patients with and without ICUAW. | RFcsa, RFMT,  VIMT | The best cut-off ratio of reduction in muscle parameters for diagnosing ICUAW using US is more than 15% for ΔTHday10 and more than 12% for ΔCSAday10 in the lower extremity of the right side.Changes in muscle ΔTHday10 and ΔCSAday10 of the lower extremities were found to have close diagnostic validity to SOFA and APACHE II scores at the time of ICU admission. |
| ***Arai et al.*** | To investigate whether QMLT and RFcsa were correlated with CT measurementsand could identify patients with low muscularity at ICU admission. | QMLT, RFcsa | The US measurements of the QMLT and RF-CSA can serve as indicators of low muscularity at ICU admission. The QMLT of 2.0 cm and the RFcsa of 4.7 cm2 can be set as cut-off values for low muscularity at ICU admission. |
| ***Hernández-Socorro et al.*** | To analyze novel US muscle assessment methods in mechanically ventilated, long-stay ICU patients. | RFcsa, RFMT | Novel sonographic muscle tools can be used to assess the muscle quantity and quality wasting process in this specific group of critically ill patients and should, due to their clinical relevance, be added to sonographic musculoskeletal diagnostic protocols. |
| ***Passos et al.*** | To correlate the nutritional status of ICU patients with the 28-day mortality rate,  to establish cut-off values for USRFcsa for the classification of the nutritional status of critically ill patients. | RFcsa | It was demonstrated that the impairment of patients' nutritional status on ICU admission is correlated with their prognosis. It has been demonstrated that value for RFcsa, as assessed by US in the first 48 h of ICU admission, lower than or equal to the cut-off value was the only independent predictor for mortality at 28 days. |
| ***Mendes et al.*** | To evaluate short‐term muscle loss in ICU patients. | RFMT | US assessment detected muscle mass loss in the short‐term more sensitively than the anthropometric method. Patients with lower RFMT on admission had a higher mortality. Malnourished and eutrophic patients are more susceptible to muscle loss than overweight patients. |
| ***Formenti et al.*** | To assess if the quality characteristics of parasternal intercostal, diaphragm and quadriceps muscles of ICU COVID19 mechanically ventilated patients influenced the outcomes. | RFcsa, RFMT | Early changes in the muscle parameters may potentially predict the survival rate. Echogenicity of RF is higher in non-survivors, and correlates better with indices of catabolism as compared with muscle thickness or CSA. Greyscale analysis of both respiratory and peripheral muscles could be a better ultrasound predictor of outcome. |
| ***Maskos et al.*** | To evaluate the utility of temporalis muscle thickness compared to RFMT in monitoring muscle wasting during the stay in a NICU,  to compare US and CT-basedmeasurements in their interrater reliability. | RFMT | TMT and RFMT showed comparable results in sequential measurements and high inter-rater reliability in both CT and US measurements; the decline in thickness correlated significantly between both muscles. |
| ***Anand et al.*** | To assess the correlation between the percentage longitudinal change of anterior temporalis MLT over 7 days with the corresponding changes in QMLT,  to assess the relation of US parameters with ICU LOS and mortality. | QMLT | Measurement of US MLT and grayscale parameters of anterior temporalis muscle do not show good correlation with that of quadriceps muscle. Serial US measurements of anterior temporalis muscle may not be clinically useful in monitoring muscle wasting in critically ill patients with sepsis. No significant correlation between ICU LOS and baseline thicknesses of either muscle groups. |
| ***Formenti et al.*** | To describe the time-course of muscular US features of both diaphragm and RF,  to determine muscular US feature possible correlation with BIVA parameters and patient outcome. | RFcsa | Body composition is significantly modified after one week of ICU stay, both in terms of BIVA parameters and in terms of muscle ultrasonography. Both diaphragmatic thickness and RFcsa are significantly reduced during the first week of ICU stay. BIVA parameters and muscle ultrasound results are not related to each other. Only diaphragma ticthickness is correlated with ICU mortality. |
| ***Formenti et al.*** | To evaluate peripheral and respiratory muscle ultrasound characteristics and their variations during a long-term ICU care. | RFcsa | 57% of the patients enrolled developed ICUAW, regardless of age, sex, diagnosis or severity at ICU admission. Patients who developed ICUAW experienced- longer ICU LOS and a trend towards a higher mortality rate. The RFcsa decreased significantly over the first week of ICU stay. The reduction rate of the pennation angle of RF was greater in patients who developed ICUAW, and this proved to be the best predictor of ICUAW, as compared to all the other variables tested. |
| ***Kangalgil et al.*** | To assess changes in muscle mass and anthropometric measurement in critically ill trauma and surgical patients,  to assess associations between muscle loss, changes in anthropometric measurements, and 90-day mortality. | RFcsa | This study confirmed prior studies demonstrating acute rapid muscle loss within the first week of ICU admission.There was no association between RFcsa and 90-day mortality. |
| ***Wu et al.*** | To determine the muscle atrophy curves of different muscle types in SICU patients. | RFMT,  VIMT | The rate of atrophy of the RF in the first month in the ICU was 0.84% per day and that of VI was 0.98%, with the highest atrophy rates in the 3rd and 4th weeks compared to the first 2 weeks. The rate of muscle atrophy in women was approximately three times higher than that in men, which suggests that more attention must be paid to women in terms of prevention and treatment of muscle atrophy. The older the person, the faster was the loss of muscle thickness. |
| ***Chapple et al.*** | To determinewhether the optimizing of nutrition therapy attenuated acute skeletal muscle loss and maintained strength and function when measured objectively across the course of recovery when compared to routine care. | QMLT | Delivering greater amounts of calories to critically ill patients whilst in ICU may not influence muscle size or strength during the hospital admission or muscle size at 3- or 6- months. Augmented calorie delivery had no quantifiable effect on quadriceps, forearm, or mid-upper arm muscle thickness or handgrip strength during the hospital admission; did not influence muscle size at 3- and 6-months after randomization. |
| ***Hrdy et al.*** | To assess the incidence ofloss of muscle mass in ICU patients,  to identify any predictors for muscle wasting. | RFcsa | Patient age at admission was associated with a risk of developing clinically significant muscle wasting. Mortality rates were two-fold higher in patients with significant muscle wasting (≥ 10% over the first seven days in the ICU) than among those without significant wasting, though this difference in mortality was not statistically significant. |

Abbreviations: BIVA: Bioelectrical Impedance Vector Analysis; CFS: clinical fraility score; ESLD: end-stage liver disease; EPA: eicosapentaenoic acid; EMS: electrical muscle stimulation; HHD: hand-held dynamometry; HMB: beta-hydroxy-beta-methylbutyrate; ICU: intensive care unit; ICUAW: intensive care unit acquired weakness; LOS: length of stay; MC: maximum compressed; Mnutric: modified nutrition risk in critically ill; MLT: muscle layer thickness; MRC: medical research council; NED: neuromuscular disorder; NEMS: neuroelectrical muscle stimulation; NICU: neurointensive care unit; QMcsa: quadriceps muscle cross sectional area; QMLT: quadriceps muscle layer thickness; RF: rectus femoris; RFcsa: rectus femoris cross sectional area; RFMT: rectus femoris muscle thickness; SICU: surgical intensive care unit; TAcsa: tibialis anterioris cross sectional area; TAMT: tibialis anterioris muscle thickness; TBI: traumatic brain injury; TMT: temporalis muscle thickness; US: ultrasound; VIcsa: vastus intermedius cross sectional area; VIMT: vastus intermedius muscle thickness; VLcsa: vastus lateralis cross sectional area; VLMT: vastus lateralis muscle thickness.

**Table s4 – Tibialis Anterior Cross Sectional Area: summary of ultrasound methods employed in each included study for the measurements of TA-CSA and its values at baseline, short and long follow-up.**

| Author | Transducer | Patient Position | Level | Compression | Side | Baseline  (D0-D3) cm^2^ | Short FU  (≤ D7) cm^2^ | Long FU  (> D7) cm^2^ | Note |
| --- | --- | --- | --- | --- | --- | --- | --- | --- | --- |
| *Annetta et al.* | 5-7.5 MHz linear | Supine, both legs in passive extension | 5 cm below peroneal head | Min | Both | 5.50 ± 1.46 | 4.70 ± 1.46 | 4.10 ± 1.00 | Two measurements in each leg; with lower limb fractures, measurements were taken on the contralateral leg only |
| *Connolly et al.* | 2–6 MHz, curvilinear | Semisupine, knee in passive extension, neutral foot | 1/3 TP - LM | Min | Right | 8.66 ± 2.76 | 8.14 ± 2.34 | NA |  |
| *Mayer et al.* | 8.5 MHz, linear | NA | 1/3 lateral TP - inferior LM border | Min | Right | 5.28 ± 0.89 | 4.71 ± 0.95 | NA | Average value of 3 consecutive measurements |

Note: Data reported as mean ± SD, median [IQR]. All measurements are expressed in cm^2^; if studies have expressed data in different units, these are specified in the table.

Abbreviations: D: days of ICU stay; FU: follow-up; LM: lateral malleolus; TP: tibial plateau; Max: maximum; Min: minimum; NA: not available.

**Table s5 – Tibialis Anterior Thickness: summary of ultrasound methods employed in each included study for the measurements of TA thickness and its values at baseline, short and long follow-up.**

| Author | Transducer | Patient Position | Level | Compression | Side | Baseline  (D0-D3) cm | Short FU  (≤ D7) cm | Long FU  (> D7) cm | Note |
| --- | --- | --- | --- | --- | --- | --- | --- | --- | --- |
| *Cartwright et al.* | 18 MHz, linear | NA | 5 cm distal to the fibular head | Min | NA | 2.14 ± NA | 2.33 ± NA | 2.40 ± NA |  |
| *Annetta et al.* | 5-7.5 MHz, linear | Supine. Both legs in passive extension | 5 cm below the peroneal head | Min | Both | 2.23 ± 0.39 | 2.10 ± 0.31 | 1.80 ± 0.31 | Two measurements in each leg; with lower limb fractures, measurements on the contralateral leg only |
| *Silva et al.* | 7.5 MHz | NA | 1/4 IBP-LM | Min | NA | NA | NA | NA | Average value of 3 measurements |
| *Silva et al.* | 7.5 MHz, linear | NA | 1/4 IBP-LM | Min | NA | NA | NA | NA | Average value of 3 measurements |
| *Mayer et al.* | 8.5-MHz, linear | NA | 1/3 LTP-IBLM | Min | Right | 2.01 ± 0.36 | 1.82 ± 0.31 | NA | Average value of 3 measurements |
| *Baston et al.* | 3-12 MHz, linear | Ankle and knee positioned in neutral extension, knee oriented vertically | 10 cm distal to the tibial tubercle | Min | NA | NA | NA | NA | A point 0.5 cm distant from the interosseous membrane’s insertion on the tibia was measured: from this point muscle linear depth was measured to the muscle-subcutaneous tissue interface keeping the measure parallel to the tibial plane |

Note: Data reported as mean ± SD. All measurements are expressed in cm; if studies have expressed data in different units, these are specified in the table.

Abbreviations: D: days of ICU stay; FU: follow-up; IBLM: inferior border lateral malleolus; IBP: inferior border patella; LM: lateral malleolus; LTP: lateral tibial plateau; Max: maximum; Min: minimum; NA: not available

**Table s6 – Vastus Intermedius Thickness: summary of ultrasound methods employed in each included study for the measurements of VI thickness and its values at baseline, short and long follow-up**

| Author | Transducer | Patient Position | Level | Compression | Side | Baseline  (D0-D3) cm | Short FU  (≤ D7) cm | Long FU  (> D7) cm | Note |
| --- | --- | --- | --- | --- | --- | --- | --- | --- | --- |
| *Gerovasili et al.* | 7.5 MHz, linear | Supine. Legs lying flat in extension | 1/2 ASIS-MPP | Min | Both | EMS:  Right 0.91 ± 0.39 Left 0.86 ± 0.36  Control:  Right 1.40 ± 0.64 Left 1.53 ± 0.67 | EMS:  Right 0.81 ± 0.38 Left 0.77 ± 0.35  Control:  Right 1.11 ± 0.56 Left 1.31 ± 0.65 | NA |  |
| *Parry et al.* | 8.5-MHz, linear | Supine. Knee passive extension, neutral rotation | 2/3 ASIS-SPB | Min | NA | 1.91 ± 0.73 | NA | NA | Average of 3 measurements |
| *Hayes et al.* | 6–15 MHz, linear | Supine. Pillow under the head; hip and knee extended; leg in neutral rotation | 2/3 ASIS-SPB | Min | Non-cannulated leg; both cannulated: that with the venous cannula (vaECMO) or with better access to the thigh (vvECMO) | 0.90 ± 0.30 | NA | 0.70 ± 0.20 | Sagittal plane, average of 3 measures |
| *Nickels et al.* | NA | NA | 2/3 ASIS-SPB | NA | Right thigh, unless inaccessible | NA | NA | NA | Average of 3 measurements |
| *Zhang et al.* | 10–13 Hz, linear | Supine. Extended knees and relaxed muscles, toes pointing to the ceiling | 1/2 AIIS-SPB | Min | Both | NA | NA | NA | Averageof 3 measurementswithin 10% |
| *Wu et al.* | 8 MHz, Linear | Supine. Right footextended | 1/2 ASIS - SPB | Min | Right | 1.34 ± 0.57 | NA | NA |  |

Note: Data reported as mean±SD. All measurements are expressed in cm; if studies have expressed data in different units, these are specified in the table.

Abbreviations: AIIS: anterior inferior iliac spine; ASIS: anterior superior iliac spine; D: days of ICU stay; ECMO: extracorporeal membrane oxygenation; EMS: electrical muscle stimulation; FU: follow-up; Max: maximum; Min: minimum; MPP: midpoint of the patella; NA: not available; SPB: superior patella border.

**Table s7 – Vastus Lateralis Thickness: summary of ultrasound methods employed in each included study for the measurements of VL thickness and its values at baseline, short and long follow-up**

| Author | Transducer | Patient Position | Level | Compression | Side | Baseline (D0-D3) cm | Short FU  (≤ D7) cm | Long FU  (> D7) cm | Note |
| --- | --- | --- | --- | --- | --- | --- | --- | --- | --- |
| *Parry et al.* | 8.5-MHz, linear | Supine. Knee in passive extension and neutral rotation | 2/3 ASIS-SPB | Min | NA | 2.31 ± 0.57 | NA | NA | Average of 3 measurements |
| *Turton et al.* | Linear | 45°, lower limb flat, externally rotated, probe handle parallel to the ground | 10 cm proximal to the lateral femur condyle, in plane, to image both the superficial and deep aponeuroses of the VL | NA | Right | 1.53 ± 0.77 | 1.40 ± 0.46 | 1.18 ± 0.36 |  |
| *Hayes et al.* | 6–15 MHz, linear | Supine.Pillow under the head; hip and knee extended; leg in neutral rotation | 2/3 ASIS-SPB | Min | Non-cannulated leg; both cannulated: that with the venous cannula (vaECMO) or with better access to the thigh (vvECMO) | 1.60 ± 0.30 | NA | 1.10 ± 0.30 | Sagittal plane, average of 3 measurements |

Note: Data reported as mean±SD. All measurements are expressed in cm. If studies have expressed data in different units, these are specified in the table.

Abbreviations: ASIS: anterior superior iliac spine; D: days of ICU stay; ECMO: extracorporeal membrane oxygenation; FU: follow-up; SPB: superior patella border; Max: maximum; Min: minimum; NA: not available.

**Table s8 –Cross Sectional Area of the entire Quadriceps Muscle: summary of ultrasound methods employed in each included study for the measurements of QM-CSA and its values at baseline, short and long follow-up.**

| Author | Transducer | Patient Position | Level | Compression | Side | Baseline  (D0-D3) cm^2^ | Short FU  (≤ D7) cm^2^ | Long FU  (> D7) cm^2^ | Note |
| --- | --- | --- | --- | --- | --- | --- | --- | --- | --- |
| *Viana et al.* | 5-12 MHz, linear | NA | 4, 10, 16 cm cranial to SPB | NA | Right or left (following local accessibility) | NA | HMB treatment:  110.50 ± 120.63  Placebo:  114.0 ± 128.93 | HMB treatment:  99.32 ± 132.92  Placebo:  100.40 ± 122.43 | sum of the 3 surfaces measured at 4, 10, 16 cm cranial to the SPB |

Note: Data reported as mean±SD. All measurements are expressed in cm^2^. If studies have expressed data in different units, these are specified in the table.

Abbreviations: D: days of ICU stay; FU: follow-up; HMB: beta-hydroxy-beta-methylbutyrate; SPB: superior patella border; NA: not available.

**Table s9 – Intra and inter – observer reliability coefficient**

| Author | Intra or Interobserver | Intraclass Correlation Coefficient |
| --- | --- | --- |
| *Quadriceps Muscle Layer Thickness (QMLT)* | | |
| *Baldwin et al.* | Intra-observer | *na* |
|  | Inter-observer | 0.976 |
| *Sarwal et al.* | Intra-observer | *na* |
|  | Inter-observer | 0.91 (95%CI 0.79-0.96) |
| *Francis et al.* | Intra-observer | *na* |
|  | Inter-observer | 0.99 |
| *Paris et al.* | Intra-observer | 0.98 |
|  | Inter-observer | 0.94 |
| *Prado et al.* | Intra-observer | - 1/2: 0.74 (95%CI 0.63-0.84) - 2/3: 0.83 (95%CI 0.75-0.90) |
|  | Inter-observer | - 1/2: 0.76 (95%CI 0.66-0.86) - 2/3: 0.83 (95%CI 0.75-0.90) |
| *Silva et al.* | Intra-observer | 0.99 |
|  | Inter-observer | *na* |
| *Dimopoulos et al.* | Intra-observer | 0.99 (95%CI 0.97-0.99) |
|  | Inter-observer | *na* |
| *Tourel et al.* | Intra-observer | 0.98 |
|  | Inter-observer | 0.96 |
| *Yanagi et al.* | Intra-observer | *na* |
|  | Inter-observer | 0.951 |
| *Baston et al.* | Intra-observer | *na* |
|  | Inter-observer | - 10cm: 0.98 (95%CI 0.92-0.99) - 20cm: 0.99 (95%CI 0.97-1.00) |
| *Lambell et al.* | Intra-observer | 0.99 |
|  | Inter-observer | 0.99 |
| *Rodrigues et al.* | Intra-observer | range: 0.94-0.99 |
|  | Inter-observer | range: 0.94-0.99 |
| *Toledo et al.* | Intra-observer | *na* |
|  | Inter-observer | 0.90 |
| *Rectus Femoris cross sectional area (RF CSA)* | | |
| *Mueller et al.* | Intra-observer | *na* |
|  | Inter-observer | 0.96 |
| *Hayes et al.* | Intra-observer | 0.98 (95%CI 0.92-0.99) |
|  | Inter-observer | *na* |
| *Borges et al.* | Intra-observer | *na* |
|  | Inter-observer | 0.97 (95%CI 0.90-0.99) |
| *Pita et al.* | Intra-observer | 0.95 |
|  | Inter-observer | *na* |
| *McNelly et al.* | Intra-observer | 0.9 |
|  | Inter-observer | *na* |
| *Nakanishi et al.* | Intra-observer | 0.99 |
|  | Inter-observer | 0.99 |
| *Rectus Femoris Muscle Thickness (RF MT)* | | |
| *Silva et al.* | Intra-observer | 0.98 |
|  | Inter-observer | *na* |
| *Zhang et al.* | Intra-observer | *na* |
|  | Inter-observer | 0.9 |
| *Mendes et al.* | Intra-observer | 0.982 |
|  | Inter-observer | *na* |
| *Maskos et al.* | Intra-observer | *na* |
|  | Inter-observer | 0.79 (95%CI 0.74-0.84) |
| *Wu et al.* | Intra-observer | 0.98 |
|  | Inter-observer | *na* |
| *Tibialis Anterior Muscle Thickness (TA MT)* | | |
| *Silva et al.* | Intra-observer | 0.99 |
|  | Inter-observer | *na* |
| *Baston et al.* | Intra-observer | *na* |
|  | Inter-observer | 0.97 (95%CI 0.90-0.99) |

na: not available; 95%CI: 95% Confidence interval

**Figure S1 - Forest plot of the average value of muscle mass for the three most frequently reported ultrasound methods (Left panel: Rectus femoris CSA, Middle panel: Quadriceps muscle layer thickness, Right panel: Rectus femoris MT) at baseline and at the shortest and longest follow-up.**


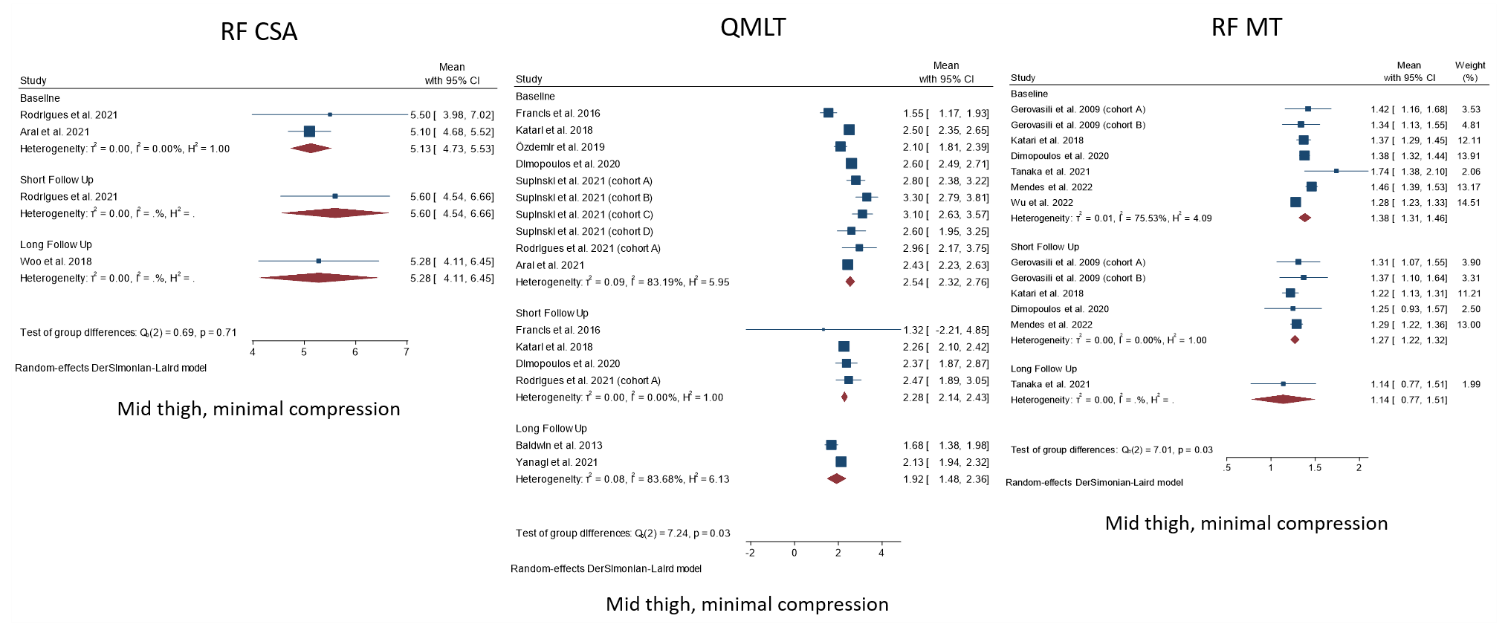


The figure reports the value of muscle mass assessment using no or minimal compression and a mid thigh landmark.

**Figure S2 - Forest plot of the average value of muscle mass for the Quadriceps muscle layer thickness at baseline and at the shortest and longest follow-up with different degrees of probe compression and different anatomic landmark.**


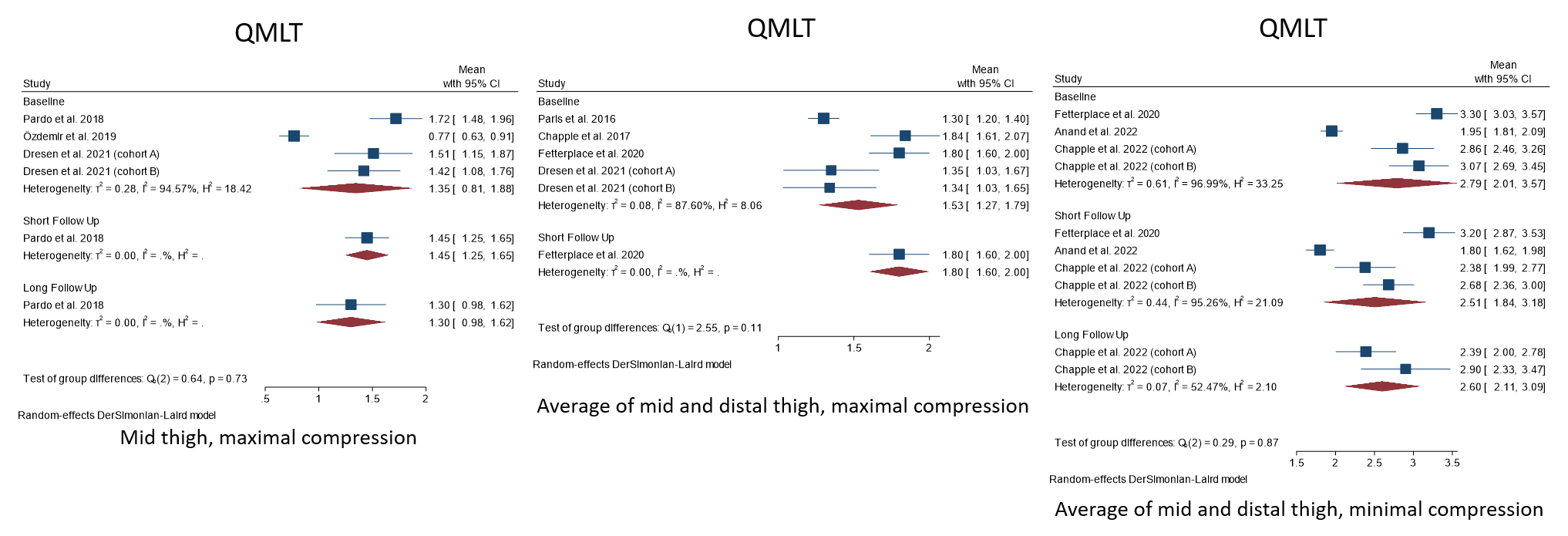


**Figure S3 - Forest plot of the average value of muscle mass for the Vastus Intermedius (left panel), Vastus Lateralis (middle panel) and Tibialis Anterior (right panel) muscle thickness at baseline and at the shortest and longest follow-up.**


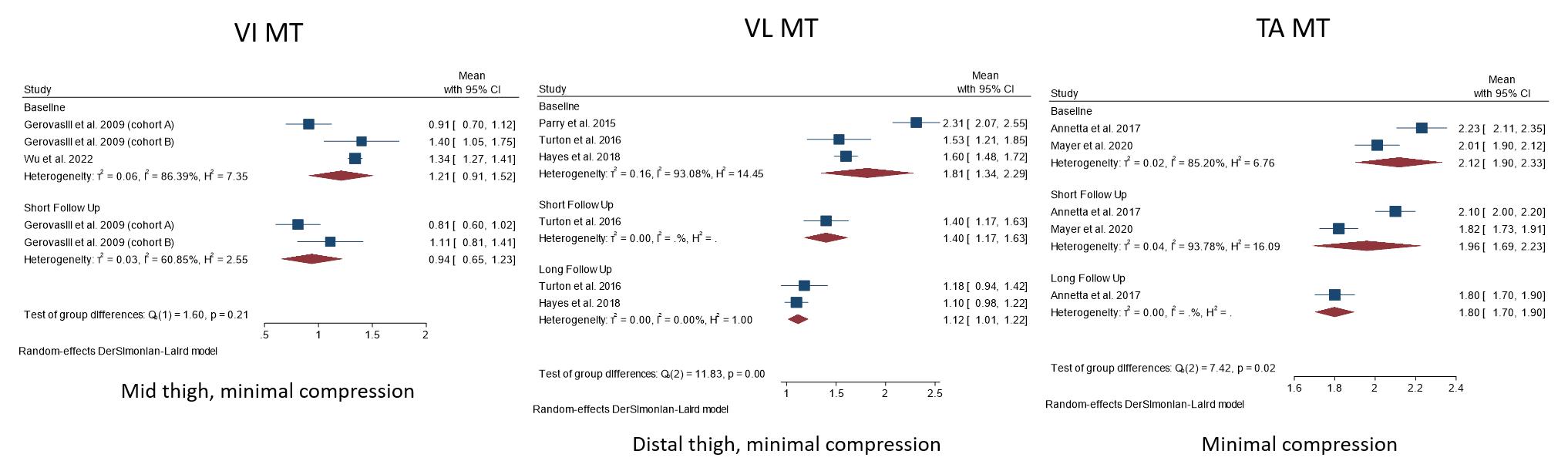

Supplement: Supplementary file 1 — Supplementary Material 1 [file 13613_2024_1395_MOESM1_ESM.docx]
